# Cyanopyridinium-Based Ionic Liquids and Their Mixtures for Ethylene and Ethane Separation

**DOI:** 10.1021/acssuschemeng.5c01481

**Published:** 2025-07-24

**Authors:** Sam H. McCalmont, Guillaume Simon, H. Q. Nimal Gunaratne, Margarida Costa Gomes, David M. Wilkins, John D. Holbrey, Leila Moura

**Affiliations:** † QUILL Research Centre, School of Chemistry and Chemical Engineering, 1596 Queen’s University Belfast, Belfast BT9 5AG, Northern Ireland, U.K.; ‡ Laboratoire de Chimie de l’ENS Lyon, 26911 CNRS and Université de Lyon, 46 Allée d’Italie, 69364 Lyon, France; § Centre for Quantum Materials and Technologies, School of Mathematics and Physics, Queen’s University Belfast, Belfast BT7 1NN, Northern Ireland, U.K.

**Keywords:** ionic liquids, cyanopyridinium, ethane, ethylene, hydrocarbon separation, gas solubility, charge-transfer complex

## Abstract

The solubility of ethane and ethylene was determined in a series of cyanopyridinium ionic liquids known to form charge-transfer complexes with polyaromatic hydrocarbons to determine their potential to form specific interactions with the unsaturated gas. The solubilities of ethylene and ethane in 1-butyl-4-cyanopyridinium bis­(trifluoromethane)­sulfonimide ([C_4_^4^CNPy]­[NTf_2_]) and 1-butyl-3-cyanopyridinium bis­(trifluoromethane)­sulfonimide ([C_4_^3^CNPy]­[NTf_2_]) were measured using an isochoric saturation method. The mole fraction solubility of ethane in the ionic liquids ranged from 6.0 × 10^–3^ to 7.2 × 10^–3^ and from 7.5 × 10^–3^ to 9.9 × 10^–3^ for ethylene in [C_4_^3^CNPy]­[NTf_2_] and [C_4_^4^CNPy]­[NTf_2_] at 0.1 MPa and 313 K, respectively. The small preferential solubility of ethylene over ethane in the ionic liquids results in ideal ethylene separation selectivities between 1.2 and 1.4, which is in the same range as typical physisorbent ionic liquids of the same type and molecular weight, indicating that there is no significant preferential interaction between the ionic liquids and ethylene. The calculated thermodynamic properties of solvation reveal that the solvation of both gases is entropically driven. To promote cyanopyridinium–ethylene interactions and decrease the possibility of steric constrictions to the interactions, 1-butyl-4-methylimidazolium bis­(trifluoromethane)­sulfonimide ([C_4_C_1_Im]­[NTf_2_]) was added as diluent to [C_4_^4^CNPy]­[NTf_2_]. This IL mixture was found to behave almost ideally based on isothermal titration nanocalorimetry results. The solubility of ethylene or ethane in the mixture was found to be the weighted average of the corresponding solubilities in the two pure ionic liquids, still indicating that no specific ethylene–ionic liquid interactions were formed. Molecular dynamics (MD) simulations of the systems were performed and revealed that the slightly higher ethylene solubility in [C_4_^4^CNPy]­[NTf_2_] is due to a slightly stronger association with this cation compared to the 3-isomer.

## Graphical abstract

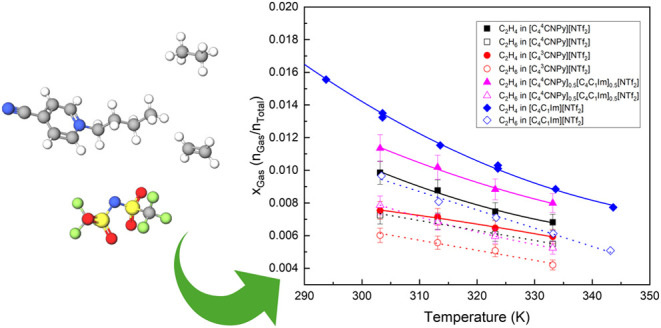

## Introduction

Separation processes contribute to around 10–15% of global energy consumption.^1,2^ Industrial separation of ethane/ethylene is performed via cryogenic distillation, an energy-intensive process that accounts for roughly 0.3% of the world’s energy demand, with corresponding greenhouse gas emissions.^2,3^ This large energy requirement is related to a combination of the high olefin gas purity requirements, the similar size and properties of the gases, and the required tonnage, over 250 million annually and increasing.^4^ Substantial research has been dedicated to the discovery of alternative materials and technologies that could help provide sustainable and low-energy alternatives to cryogenic distillation. These include selective absorption by porous materials including activated carbons,^5,6^ zeolites,^7,8^ molecular frameworks,^9–15^ polymers,^16^ selective transport membranes,^17–22^ and ionic liquids.^23,24^ Two main approaches are taken in the design of these materials: Either size-based separation or ethylene complexation via a reactive metal center, typically in the form of silver or copper­(II) ions. Size-based separation of ethylene/ethane leads to low selectivities, typically not exceeding 10, due to the size similarities of the two gases.^25^ Despite providing selectivities several times higher than the size-based approach, the use of metal-based systems has drawbacks such as the irreversible reaction of the metals with common hydrocarbon stream contaminants, including alkynes that can form explosive acetylides; sulfur compounds that are able to produce insoluble metal sulfides and hydrogen; and reduction and plating of metals from materials. This can also be photoinduced, particularly for silver ions in the presence of ambient light.^3^

Ionic liquids (ILs) have been investigated as potential adsorbents for ethylene and ethane separations, taking advantage of the potential to fine-tune their properties by using different combinations of anions and cations and their negligible volatility that avoids gas stream contamination and facilitates absorbent regeneration.

It has been previously observed that for purely physisorbent ILs, the largest hydrocarbon gas solubilities were found in phosphorus-based ILs such as tetradecyl­(trihexyl)­phosphonium bis­(2,4,4-trimethylpentyl)­phosphinate, [P_6,6,6,14_]­[DiOP], which has Henry’s law constants, *K*_H_, of 19 and 36 bar at 313 K for ethane and ethylene, respectively. However, the ideal separation selectivity for ethylene over ethane in this IL is also among the lowest reported, at 0.5.^23,26,27^ In contrast, 1-(3-cyanopropyl)-3-methylimidazolium dicyanamide, [C_3_C_1_CNIm]­[DCA], displays the highest selectivity of 2.1, but has much poorer absorption capacities with *K*_H_ of 357 and 757 bar for ethane and ethylene, respectively.^28^ In a simple binary separation of ethane and ethylene, both [P_6,6,6,14_]­[DiOP] and [C_3_C_1_CNIm]­[DCA] could be considered the best current IL candidates for this separation, the first targeting the saturated gas and the second the unsaturated gas, with the first presenting a greater overall gas uptake capacity.

Moura et al. observed that C2 and C3 hydrocarbon absorption characteristics are similar across a wide variety of ILs. The inverse relationship between uptake capacity, selectivity, and IL molecular weight is an indication of the weak gas–liquid interactions at play in the solvation of these gases. Specifically, the solubility of the saturated gas increases faster than that of the corresponding unsaturated one with the increase of the size of the nonpolar domains in either the cations or anions of the ILs. Generally, the gas solubilities in ILs follow the cation order of [P*_nmpq_*]^+^ > [C*_n_* Pyr]^+^ > [C*_n_* C*_m_* Pyrr]^+^ > [C*_n_* C*_m_* I*m*]^+^.^23^

To increase capacity and selectivity for unsaturated hydrocarbons, more specific gas–ionic liquid interactions have to be promoted, as has been observed for ionic liquids containing terminal nitrile-functionalized alkyl groups that exhibit enhanced selectivity toward olefins.^28^

In this work, we have investigated whether the incorporation of nitrile groups within the cationic core of ionic liquids could provide a vehicle for enhancement in either olefin capacity or selectivity. Cyanopyridinium cations were investigated due to their ability to form stabilized ionic liquid-aromatic 1:1 charge-transfer complexes with electron-rich π-aromatic hydrocarbons, such as 1-methylnaphthalene, and the investigations here explore whether analogous potential π-cation association with olefins would result in increased ethylene solubility and selectivity over ethane compared to analogous ILs without nitrile functionality.^29–31^

To do this, we have measured the solubility of ethane, ethylene, and a mixture of the gases in three IL systems, 1-butyl-4-cyanopyridinium bis­(trifluoromethyl)­sulfonylimide ([C_4_^4^CNPy]­[NTf_2_]), 1-butyl-3-cyanopyridinium bis­(trifluoromethyl)­sulfonylimide ([C_4_^3^CNPy]­[NTf_2_]), and mixtures of [C_4_^4^CNPy]­[NTf_2_] with 1-butyl-3-imidazolium bis­(trifluoromethane)­sulfonimide ([C_4_C_1_Im]­[NTf_2_]) using an isochoric saturation method as a function of temperature and pressure. The ideal and real selectivities of the absorbents toward the uptake of ethylene along with the thermodynamic properties of solvation were calculated from the solubility results. The IL mixtures ([C_4_^4^CNPy]­[NTf_2_] and [C_4_C_1_Im]­[NTf_2_]) were studied using isothermal titration nanocalorimetry to assess the possible interactions between the two different cations in the ionic liquid mixture and understand how these could affect the gas solubility results. Nuclear magnetic resonance (NMR) studies and molecular dynamics (MD) simulations complemented these results and aided the understanding of the solubility results at a molecular level by explaining the mechanisms of solvation.

## Experimental Section

### Materials

Ethylene (99% purity), ethane (99% purity), and a mixture of ethylene/ethane (50:50% molar balance) were supplied by BOC. 4-Cyanopyridine (98% purity) and 3-cyanopyridine (purity 95%) were purchased from Fluorochem. 1-Bromobutane (purity 99%) was purchased from Sigma-Aldrich, and lithium bis­(trifluoromethanesulfonyl)­imide was supplied by 3M. The ionic liquids 1-butyl-4-cyanopyridinium bis­(trifluoromethane)­sulfonimide ([C_4_^4^CNPy]­[NTf_2_]), 1-butyl-3-cyanopyridinium bis­(trifluoromethane)­sulfonimide ([C_4_^3^CNPy]­[NTf_2_]), and 1-butyl-3-imidazolium bis­(trifluoromethane)­sulfonimide ([C_4_C_1_Im]­[NTf_2_]) were synthesized following the methods described by Hardacre et al. and are shown in Figure 1. This included a metathesis step of the corresponding bromide salts, followed by water washing of the final IL until no halide was present in the washings via silver nitrate testing. The spectroscopic, thermal, and physical characterization (see below) was consistent with what was previously reported in the literature. More details on synthesis and characterization are described in the Supporting Information.

**Figure 1:**
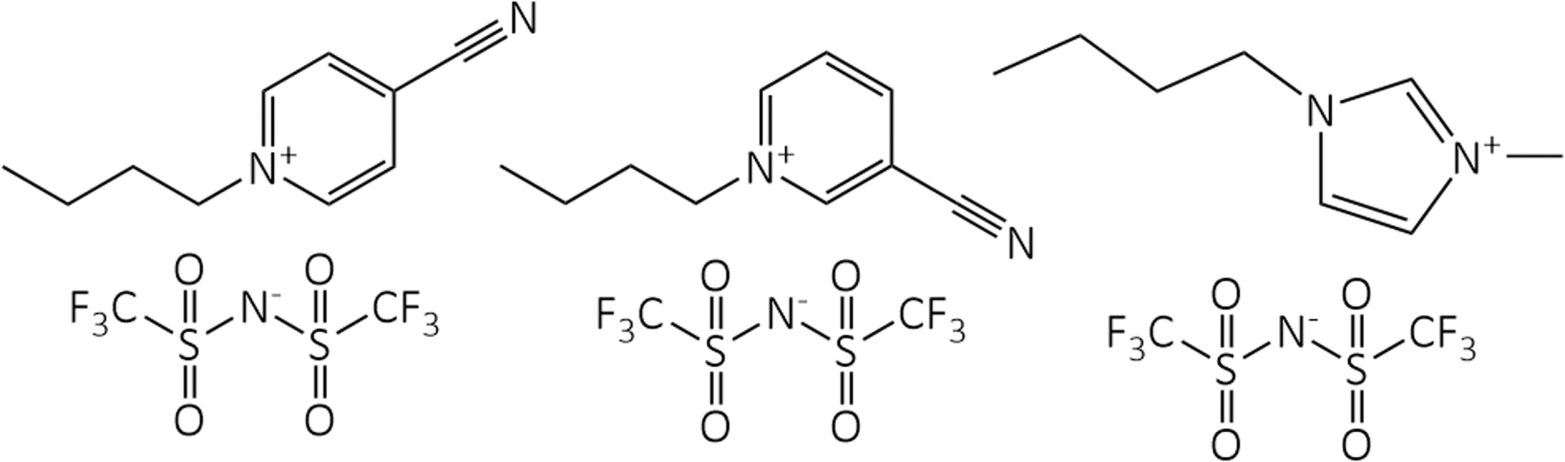
Structures of the ionic liquids used in the solubility tests with ethylene and ethane: [C_4_^4^CNPy]­[NTf_2_] (left), [C_4_^3^CNPy]­[NTf_2_] (middle), and [C_4_C_1_Im]­[NTf_2_] (right).

### Structural Characterization

#### Density and Viscosity

The densities of the ionic liquids were measured between 293 and 323 K at atmospheric pressure using an Anton Paar DMA 5000 M densimeter (ENS de Lyon) and in an Anton Paar SVM 3001 Viscometer (QUB), both calibrated with certified ultrapure water (supplied by Anton Paar). For the Anton Paar SVM 3001, the precision is estimated to be in the order of magnitude of 2.0 × 10^–5^ g cm^–3^ and the accuracy is estimated to be better than 0.01%. The temperature is controlled within ±0.005 K. For the Anton Paar DMA 500 M, the precision is estimated to be on the order of magnitude of 10^–5^ g cm^–3^ and the accuracy is estimated to be better than 0.01%. The temperature is controlled to within ±0.001 K.

Rolling ball viscosity measurements were performed with an Anton Paar Lovis 2000 ME between 293 and 343 K. The viscometer’s set of 1.8 mm capillary and stainless steel ball was calibrated prior to the measurements at 298 and 333 K between the angles 20 and 70° using the N100 standard oil reference. The accuracy and precision of the viscosity measurements were tested with N26 standard reference for viscosities in the range 11–40 mPa·s and with N415 standard reference for viscosities in the range 30–1100 mPa·s. Considering the calibration and tests performed, the accuracy and precision of the viscosity measurements are estimated to be better than 1.5 and 0.30%, respectively. The temperature was controlled to within ±0.01 K.

For the Anton Paar SVM 3001, the viscosity results are estimated to be in the order of magnitude of 0.01 mPa·s, and the accuracy is estimated to be better than 0.1%. The temperature was controlled to within ±0.005 K.

#### Thermal Analysis

The thermal behavior of the three ionic liquids was evaluated in a differential scanning calorimeter (TA Instruments, Q2000) using hermetically sealed aluminum crucibles containing ionic liquid samples of about 20 mg and a constant flow of nitrogen (50 mL min^–1^). The uncertainty associated with the TA Instruments Q2000 is ±0.1 K, and calorimetric reproducibility (with indium metal) is 0.05%. During the thermal analysis, each sample was cooled from 293 to 203 K, at a rate of 5 K min^–1^, and maintained at 203 K for 5 min. The samples were then heated to 323 K and maintained at 323 K for 5 min. This cycle was repeated 3× (each cycle started now at 323 K, instead of 293 K).

The thermal stability of the ionic liquids was evaluated in a thermogravimetric analyzer (TA Instruments, Q5000). Samples (ca. 40 mg) were placed in platinum pans and heated, under a constant nitrogen flow of 20 mL min^–1^ from 303 to 873 K at a rate of 10 K min^–1^. Weight uncertainty is 0.1 μg.

#### Water Content

The ionic liquids were dried under vacuum (100 Pa) at mild temperatures (308–318 K) for at least 24 h before the characterization. The water content in each ionic liquid after drying was determined by a coulometric Metrohm 899 Coulometer Karl Fisher titrator using Hydranal Coulmat AG-H as reagent. The water content was lower than 400 ppm for all samples; the measurements were repeated three times to form an average. Regular calibration with certified materials ensures accuracy of 5% and precision <1%.

### Gas Solubility

The measurements of gas solubility were performed in equipment built in-house in the temperature range of 303–333 K and up to 2 bar. Benchmarking and calibration of this gas solubility system (GSS) are described in the SI, and a simplified diagram of the system can be seen in Figure 2. The uncertainty of the GSS was determined as 7.26% of the output mole fraction of gas in the sorbent. The relevant uncertainties (excluding minimal uncertainties such as determining the mass and density of sorbent) occur from the pressure transmitters (0.1%), the temperature measurement in the reactor (0.5%), and the two hot plates used (6.67% total).

**Figure 2:**
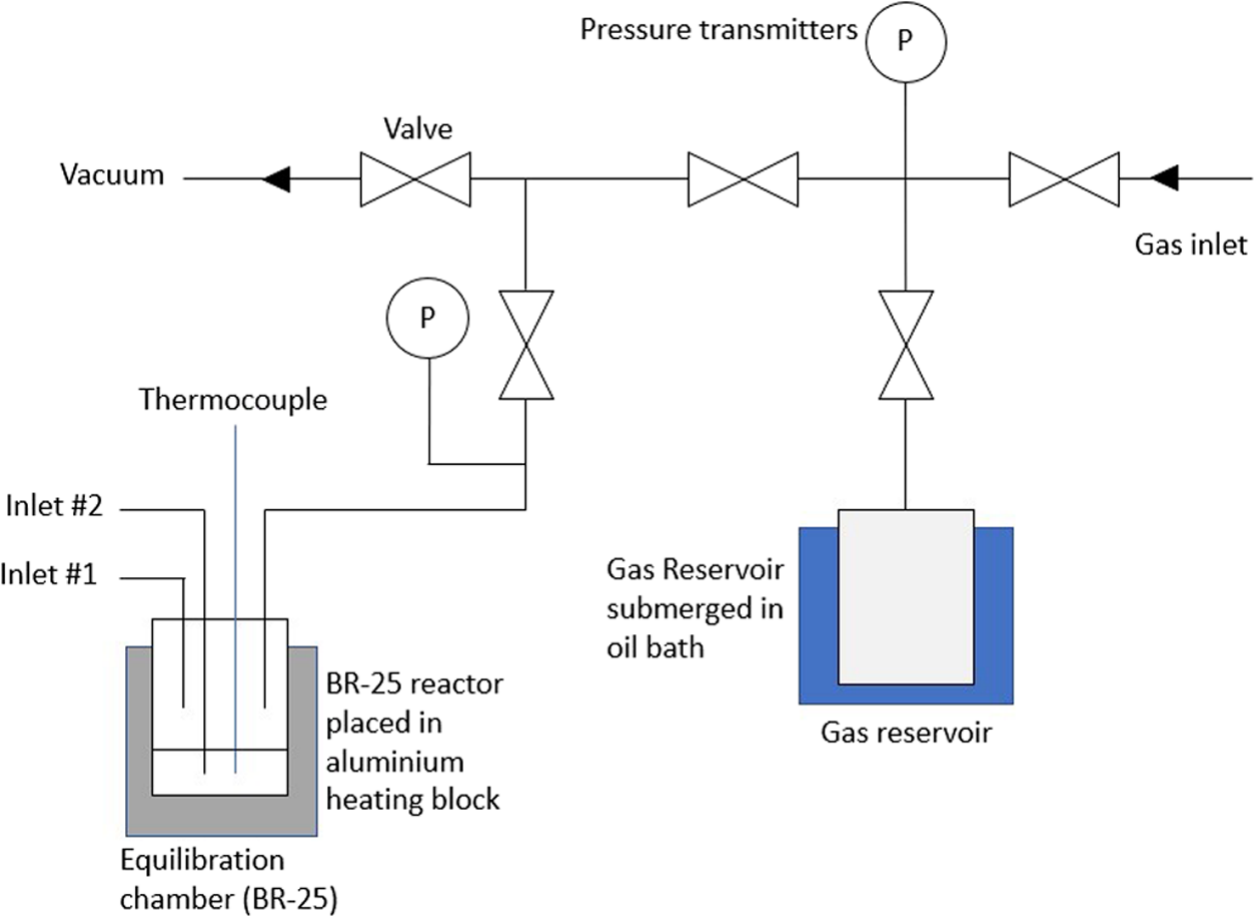
Gas solubility system (GSS) used throughout this work.

In summary, a known amount of gas, stored in the gas reservoir, is placed in contact with a known amount of degassed ionic liquid in the equilibration chamber at a set temperature. After the thermodynamic equilibrium is attained, the pVT measurements are recorded, and the temperature of the system is changed. Three measurements are performed for each gas–ionic liquid system with different samples. To calculate the gas solubility in the IL, first we determine the total amount of gas in the cell, *n*_g_^tot^, using eq 1, and the accurate measurement of pressure and temperature (*p*_ini_ and *T*_ini_) of the gas in the gas reservoir with known volume (*V*_ini_). In turn, this volume is determined via addition of degassed distilled water to the reservoir, then converting the mass of water to its respective volume using the known density at room temperature.^32^

1 $$n_{g}^{\mathrm{tot}}=\frac{p_{\mathrm{ini}}\left(V_{\mathrm{ini}}\right)}{Z\left(p_{\mathrm{ini}},T_{\mathrm{ini}}\right)RT_{\mathrm{ini}}}$$

In eq 1, *R* represents the molar gas constant and *Z* represents the compressibility factor of the gas, calculated to the level of the second virial coefficient from the data compiled by Dymond et al.^33^

After thermodynamic equilibrium between the gas and the ionic liquid is attained, measurement of the temperature and pressure is registered. The amount of nondissolved gas remaining in the cell, *n*_g_^vap^, is calculated as

2 $$n_{g}^{\mathrm{vap}}=\frac{p_{\mathrm{eq}}\left(V_{\mathrm{tot}}-V_{\mathrm{liq}}\right)}{Z\left(p_{\mathrm{eq}},T_{\mathrm{eq}}\right)\mathrm{RT}}$$

in which *p*_eq_ and *T*_eq_ are the pressure and temperature at equilibrium, respectively, and *V*_tot_ and *V*_liq_ are the total volume of the equilibration chamber and the volume occupied by the ionic liquid, respectively. In these calculations, two assumptions are made: (i) the volume of the liquid phase does not change upon dissolution of the gas and (ii) the quantity of ionic liquid in the liquid phase, *n*_IL_^liq^, remains constant as its vapor pressure is negligible in the temperature range covered. These approximations have been shown to have a negligible effect on the accuracy of the gas absorption measurements.^34,35^ The amount of gaseous solute absorbed by the ionic liquid sample, *n*_g_^liq^, is then calculated as

3 $$n_{g}^{\mathrm{liq}}=n_{g}^{\mathrm{tot}}-n_{g}^{\mathrm{vap}}$$

Gas solubilities are expressed here as the solute molar fraction, *x*_2_, and as Henry’s law constant, *K*_H_, both calculated from the amount of gas absorbed by the ionic liquid, as follows:

4 $$x_{2}=\frac{n_{g}^{\mathrm{liq}}}{n_{\mathrm{IL}}^{\mathrm{liq}}+n_{g}^{\mathrm{liq}}}$$

5 $$K_{H}=\lim_{x_{g}\to 0}\frac{\phi_{g}p}{x_{g}}$$

The calculated Henry’s law constants (*K*_H_/bar) were fitted to a power series in temperature (*T*/K), as presented in eq 6. The *A*_*i*_ coefficients are listed in the SI

6 $$\ln\left(K_{H}\right)=\sum_{i=0}^{n}A_{i}\times {\left(T\right)}^{-i}$$

The ideal selectivity is calculated as the ratio of Henry’s law constant between the two gases (ethylene and ethane) in the ionic liquid, as shown in eq 7

7 $$\alpha =\frac{K_{H,1}}{K_{H,2}}$$

where *K*_H,1_ and *K*_H,2_ represent the higher and lower Henry’s law constants from the alkane/alkene gas pair, respectively.

#### Thermodynamic Properties of Solvation

The Gibbs energy of solvation corresponds to the change in Gibbs energy when the solute is transferred, at constant temperature, from the pure perfect gas state at the standard pressure to the infinitely dilute state of the solute in the solvent and can be calculated from the experimental Henry’s law constants (eq 8)^36^

8 $$\Delta_{sol}G^{\infty }=\mathrm{RT}\ln\left(\frac{K_{H}}{p^{0}}\right)$$

The thermodynamic properties of solvation, i.e, the enthalpy of solvation, Δ_solv_ *H*, and the entropy of solvation, Δ_solv_ *S*, can provide some clarification on the molecular mechanisms determining the solution behavior. The enthalpy of solvation reflects the energy of solute–solvent interactions and the entropic contribution is related to the structural organization of the solution.^28,37^

9 $$\Delta_{sol}H^{\infty }=-T^{2}\frac{\partial }{\partial T}\left(\frac{\Delta_{sol}G^{\infty }}{T}\right)=-RT^{2}\frac{\partial }{\partial T}\left[\ln\left(\frac{K_{H}}{p^{0}}\right)\right]$$

10 $$\Delta_{\mathrm{sol}}S^{\infty }=\frac{\left(\Delta_{\mathrm{sol}}H^{\infty }-\Delta_{\mathrm{sol}}G^{\infty }\right)}{T}=-\mathrm{RT}\frac{\partial }{\partial T}\left[\ln\left(\frac{K_{H}}{p^{0}}\right)\right]-R\ln\left(\frac{K_{H}}{p^{0}}\right)$$

#### Headspace Gas Analysis

Headspace gas chromatography (HS-GC) was used to determine the composition ratio of the free gas at equilibrium during the mixed gas experiments (50:50 ethylene/ethane) solubility experiments.^38^ The gas analysis experiments were carried out using a PerkinElmer Clarus 500 gas chromatograph (GC) attached to a Turbomatrix 40 headspace (HS) autosampler using helium as a carrier and a flame ionization detector (FID) equipped with a methanizer using a nickel catalyst. The HS autosampler oven was set to equilibrate samples for 2 h at 35 °C (308 K) as the lowest possible temperature for this particular piece of equipment. The method included a high-pressure injection mode with a 40 psi injection for 2 min and a column pressure of 15 psi. The withdrawal time of sample is 6 s before the vial was vented. The needle transfer and line temperatures were set to 50 °C. The GC injector temperature was set to 50 °C, the oven to 70 °C, and the FID detector and methaniser set to 350 °C with an H_2_ flow of 45 mL min^–1^. The carrier gas (helium) pressure within the column was set to 12.5 psig and had a split flow of 10 mL min^–1^. In all experiments, the GC column used was an Agilent J&W CP-Silica PLOT with a nominal geometry of 15 m × 0.32 μm id × 4 μm df. As the gas phase and, consequently, the liquid phase are disturbed during the sampling of the gas phase for GC into a HS-GC vial, these measurements were only performed for equilibrated samples at 35 °C.

### NMR Spectroscopy

Gas–liquid interactions were probed by nuclear magnetic resonance (NMR) spectroscopy in standard 5 mm Wilmad NMR tubes. A capillary containing deuterated dimethyl sulfate (DMSO–*D*_6_) was added to the ionic liquid samples as a reference. Gases were bubbled through the neat ionic liquids using needles piercing through a thin-walled rubber septa tube cap. 1D NMR data was collected on a 400 MHz Bruker spectrometer, and two-dimensional (2D) data was collected on a 600 MHz spectrometer. ^1^H and ^13^C single-dimensional spectra were performed to confirm the existence of the gas signals and to confirm the presence of the gases in the ionic liquid.

2D spectra allowed us to evaluate the proximity of any specific gas–liquid interactions: NOESY was used for determining which signals arise from protons that are close to each other in space, even if they are not bonded; DOSY was used to separate the NMR signals of different species according to their diffusion coefficients. The change in the self-diffusion coefficients of the cation and anion of the ionic liquids before and after contact with the gases can help us understand whether gas–liquid interactions lead to a drag effect in the ions, indicating possible stronger interactions.

### Thermodynamics of Mixing

An isothermal titration nanocalorimeter (TA Instruments) equipped with a thermal activity monitor thermostat (TAM IV from TA Instruments) was used to measure the excess molar enthalpies (*H* ^E^, or mixing enthalpies, Δ*H*_mix_) of the ionic liquid mixture containing [C_4_C_1_Im]­[NTf_2_] and [C_4_^4^CNPy]­[NTf_2_] at atmospheric pressure and at 313.15 K. Two 1 mL cells (the measuring cell and the reference cell) made of Hastelloy were initially filled with similar amounts of the same liquid sample. During the titration experiments, small amounts (5, 10, 15, and 20 μL depending on the experiment) of a pure ionic liquid (either the [C_4_C_1_Im]­[NTf_2_] or [C_4_^4^CNPy]­[NTf_2_]) were injected using a 250 μL gastight Hamilton syringe into the measuring cell. The measuring cell was constantly stirred (100 rpm). A motor-driven pump (thermometric 3810 syringe pump) was used for the automatic injections (4–7 per experiment), each injection lasting 180, 300, or 480 s depending on the experiment. An interval of 60 min was allowed between injections for recovering the baseline.

After each injection, a peak corresponding to the heat effect of the mixing process is recorded. The area of the peak, *Q_i_*, is proportional to the heat involved, and it is a value required for the excess molar enthalpy calculation. Integration of the peaks recorded during the titration experiments was done by using the TAM assistant software. The consistency of the results was checked by measuring the mixing enthalpies at several mole fractions along the whole composition range.

The heat effects determined experimentally, *Q_i_*, can be related to the partial molar excess enthalpy, *H̅*_i_^E^. For example, *Q*_IL2_ corresponds to the heat effect recorded when a small amount of ionic liquid 2 (IL2), δ*n*_IL2_, is injected into ionic liquid 1 (IL1) (or into a binary mixture, IL1 + IL2, of known composition) and is related to the partial molar excess enthalpy of IL2 in the mixture, *H̅*_IL2_^E^

11 $${\mathrm{H̅}}_{\mathrm{IL}2}^{E}={\frac{\delta \left(n_{\mathrm{IL}2}+n_{\mathrm{IL}1}\right)\Delta_{\mathrm{mix}}H_{\mathrm{IL}1+\mathrm{IL}2}}{\delta n_{\mathrm{IL}2}}}_{p,T,n_{\mathrm{IL}1}}\approx \frac{Q_{\mathrm{IL}2}}{\Delta n_{\mathrm{IL}2}}$$

where *n*_IL1_ and *n*_IL2_ indicate the amounts of IL1 and IL2, respectively, and Δ_mix_ *H*_IL1+IL2_ is the enthalpy of mixing of the two ionic liquids. Δ*n*_il2_ is the quantity of IL2 per injection calculated from the injected volumes and experimental densities.

The enthalpy of mixing can be represented by the Redlich–Kister equation as a function of the mole fraction composition of the mixtures

12 $$\Delta_{\mathrm{mix}}H_{\mathrm{IL}1+\mathrm{IL}2}=\left(1-x_{\mathrm{IL}2}\right)x_{\mathrm{IL}2}\sum_{i=0}^{n}A_{i}{\left(1-2x_{\mathrm{IL}2}\right)}^{i}$$

where *x*_IL2_ denotes the mole fraction of IL2 in the mixture.

The partial molar excess enthalpy of IL2 in the mixture, *H̅*_IL2_^E^, can be obtained from the derivative with respect to the composition of eq 12 and the fitting of the Redlich–Kister parameters (*A_i_*). A more detailed description of this treatment can be found elsewhere.^39^ Finally, the enthalpy of mixing can be directly calculated from eq 12 and the partial molar excess enthalpies at infinite dilution through

13 $${\mathrm{H̅}}_{2}^{E,\infty }=\lim_{x_{2}\to 0}{\mathrm{H̅}}_{2}^{E}=\sum_{i=0}^{n}A_{i}$$

14 $${\mathrm{H̅}}_{1}^{E,\infty }=\lim_{x_{1}\to 0}{\mathrm{H̅}}_{1}^{E}=\sum_{i=0}^{n}{\left(-1\right)}^{i}A_{i}$$

### Molecular Dynamics Simulations

Molecular dynamics (MD) simulations of gas solvation in the ionic liquids [C_4_C_1_Im]­[NTf_2_], [C_4_^3^CNPy]­[NTf_2_], and [C_4_^4^CNPy]­[NTf_2_] were performed using GROMACS.^40^ For each system, 200 cations, 200 anions, and 1 molecule of ethane or ethylene were placed in a cubic box, whose side length was approximately 5 nm. These systems were equilibrated at a constant temperature of 300 K for 1 ns at constant volume and a further 2 ns at constant pressure before a production run of 700 ns at constant temperature and pressure.

For [C_4_C_1_Im]­[NTf_2_], the force field for Goloviznina et al. was used.^41^ For [C_4_^3^CNPy]­[NTf_2_] and [C_4_^4^CNPy]­[NTf_2_], we used the same Lennard-Jones and bonding parameters as we used for [C_4_C_1_Im]­[NTf_2_]. To obtain the partial atomic charges for these two cations, which have not previously been studied with the force field of Goloviznina et al.,^41^ we used the restrained electrostatic potential (RESP) method,^42,43^ in which the partial charges are fitted to give the best match with the quantum-mechanical electrostatic potential. These charges were calculated with the CP2K code at the B3LYP level.^44^ The OPLS-AA force field was used to model ethane and ethylene.^45^ Input files and equilibrated configurations are given for GROMACS, as well as an input file for CP2K, in the SI.

## Results and Discussion

### Density and Viscosity Measurements

The experimental densities of the three ionic liquids: [C_4_C_1_Im]­[NTf_2_], [C_4_^4^CNPy]­[NTf_2_], and [C_4_^3^CNPy]­[NTf_2_] and their mixtures are plotted in Figure 3, and the raw data is reported in the SI. The densities follow a decrease in the order of [C_4_^3^CNPy]­[NTf_2_] ≈[C_4_^4^CNPy]­[NTf_2_] > [C_4_^4^CNPy]_0.75_[C_4_C_1_Im]_0.25_[NTf_2_] > [C_4_^4^CNPy]_0.5_[C_4_C_1_Im]_0.5_[NTf_2_] > [C_4_^4^CNPy]_0.25_[C_4_C_1_Im]_0.75_[NTf_2_] > [C_4_C_1_Im]­[NTf_2_].

**Figure 3:**
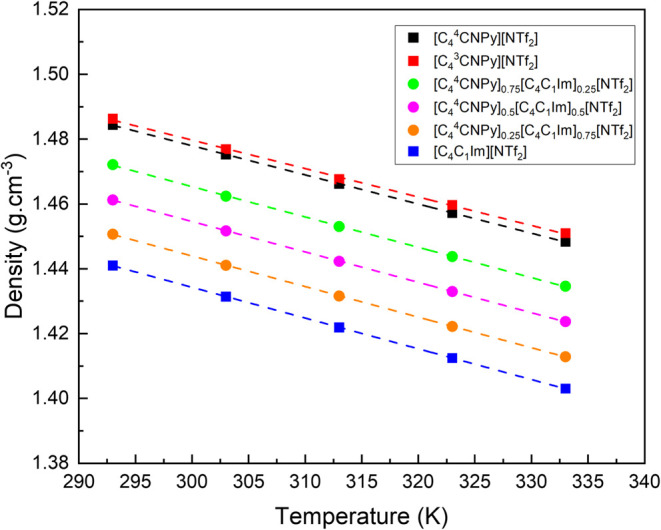
Variation of the density (g mol^–1^) with temperature for [C_4_C_1_Im]­[NTf_2_] (blue square), [C_4_^4^CNPy]­[NTf_2_] (■), and [C_4_^3^CNPy]­[NTf_2_] (red square), and the mixtures of [C_4_C_1_Im]­[NTf_2_] and [C_4_^4^CNPy]­[NTf_2_] ionic liquids: [C_4_^4^CNPy]_0.75_[C_4_C_1_Im]_0.25_[NTf_2_] (green bullet), [C_4_^4^CNPy]_0.5_[C_4_C_1_Im]_0.5_[NTf_2_] (magenta bullet), and [C_4_^4^CNPy]_0.25_[C_4_C_1_Im]_0.75_[NTf_2_] (orange bullet).

The imidazolium and the cyanopyridinium-based ionic liquids have similar densities, as they share the same anion, and have similar structure and molecular weight, with 419.36 g mol^–1^ for [C_4_C_1_Im]­[NTf_2_] and 441.37 g mol^–1^ for [C_4_^4^CNPy]­[NTf_2_] and [C_4_^3^CNPy]­[NTf_2_]. Nevertheless, [C_4_C_1_Im]­[NTf_2_] has a lower density than any of the cyanopyridinium-based ionic liquids.

The small difference in the density results of [C_4_^4^CNPy]­[NTf_2_] and [C_4_^3^CNPy]­[NTf_2_] is 0–0.2%, which is similar to the equipment’s precision level. The density of [C_4_C_1_Im]­[NTf_2_] is comparable to previously published data within the respective precision levels.^46–48^ Compared to the results obtained by Hardacre et al., the deviations range between 0.4 and 0.6% for both [C_4_^4^CNPy]­[NTf_2_] and [C_4_^3^CNPy]­[NTf_2_].^31^ Water content is not mentioned by the authors; therefore, this difference cannot be fully explained by the presence of impurities in the samples. Elementary analysis was completed for both [C_4_^4^CNPy]­[NTf_2_] and [C_4_^3^CNPy]­[NTf_2_] in Hardacre’s work along with this work (this work’s data is shown in the Supporting Analysis). For both [C_4_^4^CNPy]­[NTf_2_] and [C_4_^3^CNPy]­[NTf_2_], the compositions of carbon, hydrogen, and nitrogen were similar. No information on sulfur content was given by the authors.

The density results of [C_4_^4^CNPy]­[NTf_2_] (0.3–0.4% difference) and [C_4_^3^CNPy]­[NTf_2_] (0–1.3%) are also in similar agreement with Domańska et al.^49,50^ Domańska’s samples and this work’s share a similar water content (<500 ppm) but the authors’ NMR results indicate the presence of additional impurities that can justify the observed deviations. The comparison of elemental analysis results reveals that differences in compositions are below 3%.

The mixtures of ionic liquids present a weighted average of the components’ densities. This is to be expected since the cations involved have similar masses and structures. The [C_4_^4^CNPy]_0.5_[C_4_C_1_Im]_0.5_[NTf_2_] mixture density curve lies between both pure [C_4_C_1_Im]­[NTf_2_] and [C_4_^4^CNPy]­[NTf_2_] over the temperature range tested (see Figure 3), suggesting that there are no major changes occurring upon mixing of the ionic liquids.

The experimental viscosities of [C_4_C_1_Im]­[NTf_2_], [C_4_^4^CNPy]­[NTf_2_], and [C_4_^3^CNPy]­[NTf_2_] and their mixtures are presented in Figure 4, and the raw data is reported in the SI. The viscosities follow a similar trend to that of the density, the decreasing order being of [C_4_^3^CNPy]­[NTf_2_] > [C_4_^4^CNPy]­[NTf_2_] > [C_4_^4^CNPy]_0.75_[C_4_C_1_Im]_0.25_[NTf_2_] > [C_4_^4^CNPy]_0.5_[C_4_C_1_Im]_0.5_[NTf_2_] > [C_4_^4^CNPy]_0.25_[C_4_C_1_Im]_0.75_[NTf_2_] > [C_4_C_1_Im]­[NTf_2_]. The viscosities in the mixtures of [C_4_C_1_Im]­[NTf_2_] and [C_4_^4^CNPy]­[NTf_2_] all fall between the viscosities of both pure ionic liquids, and fall in order of increasing viscosity with increasing fraction of [C_4_^4^CNPy]­[NTf_2_].

**Figure 4:**
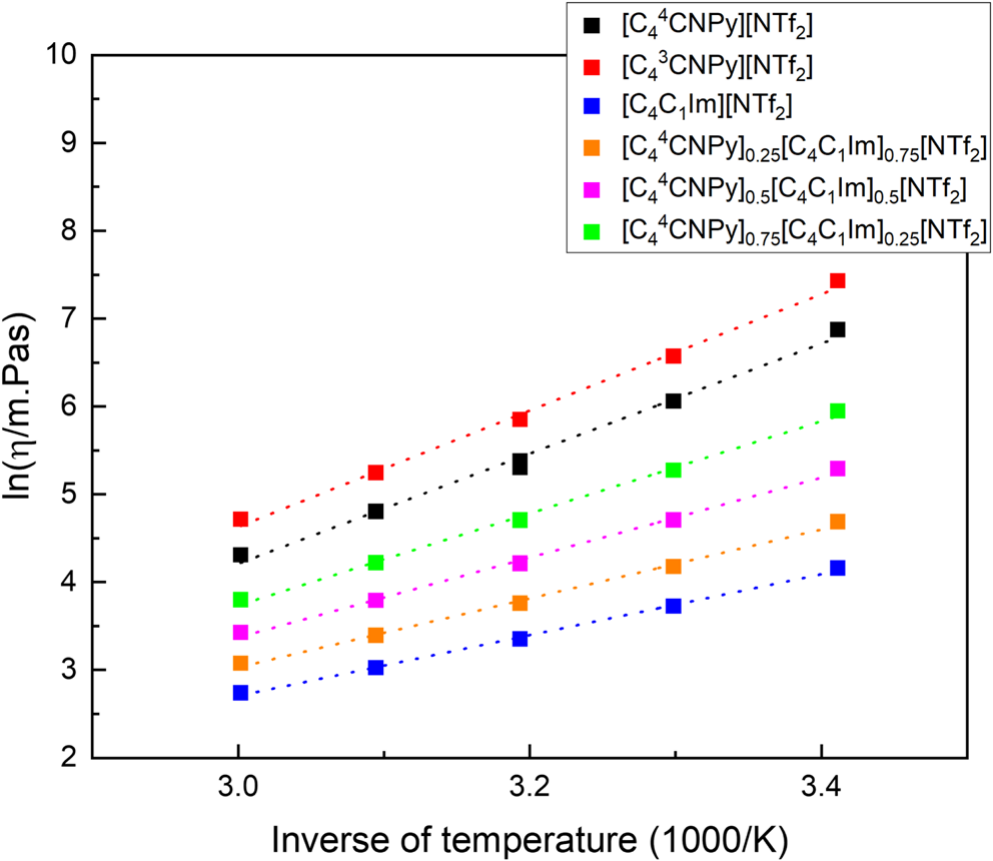
Experimental viscosity (ln­(η/mPa·s)) of the ionic liquids as a function of temperature (1/*T* (K^–1^)). The listed ionic liquids are [C_4_C_1_Im]­[NTf_2_] (blue square), [C_4_^4^CNPy]­[NTf_2_] (■) and [C_4_^3^CNPy]­[NTf_2_] (red square), and the mixtures of [C_4_C_1_Im]­[NTf_2_] and [C_4_^4^CNPy]­[NTf_2_] ionic liquids: [C_4_^4^CNPy]_0.75_[C_4_C_1_Im]_0.25_[NTf_2_] (green square), [C_4_^4^CNPy]_0.5_[C_4_C_1_Im]_0.5_[NTf_2_] (magenta square), and [C_4_^4^CNPy]_0.25_[C_4_C_1_Im]_0.75_[NTf_2_] (orange square).

All the ionic liquids used are 100 to 1000 times more viscous than water at ambient temperature, for which the viscosity is 0.89 mPa·s at 298 K.^51^ From the values adjusted to the Vogel-Fulcher-Tammann (VFT) fitting (shown in the SI), it is clear that the viscosity of the ionic liquids are of the same order of magnitude (10^–5^ Pa·s) of most molecular liquids at infinite temperature (10^–5^ to 10^–6^ Pa·s).^52^

When compared to previous publications, our viscosities present higher values, with 30–50% difference for [C_4_^4^CNPy]­[NTf_2_] and 6–20% for [C_4_^3^CNPy]­[NTf_2_], compared to those published by Hardacre et al.^31^ This difference is hard to justify since the authors do not mention the water content or impurities present in the ionic liquids. Comparing with Domańska et al. results, we observe an up to 20% difference for [C_4_^4^CNPy]­[NTf_2_] and up to 10% for [C_4_^3^CNPy]­[NTf_2_], although the authors report similar water contents in their samples.^49,50^ The viscosity of [C_4_^3^CNPy]­[NTf_2_] is between 50 and 74% larger than for [C_4_^4^CNPy]­[NTf_2_], a trend not previously reported by Hardacre.^31^ However, within their work, the data show that the 3-cyano ionic liquids are more viscous than 4-cyano, which is in agreement with this work. There could be electronic/electrostatic effects originating from the differences in the positions of the electron-withdrawing nitrile groups or topological features due to the 3-cyano ionic liquid having a larger cross section if looked down above the plane of the pyridinium ring, which could imply less close packing.

There are multiple approaches to represent the viscosities of ionic liquid mixtures. A common approach is a modified Arrhenius equation by Grunberg and Nissan^53–55^

15 $$\log\left(\mu_{m}\right)=x_{1}\log\left(\mu_{1}\right)+x_{2}\log\left(\mu_{2}\right)+x_{1}x_{2}G$$

where *x*_1_ and *x*_2_ represent the mole fractions of the two ionic liquids in the mixture, μ_1_ and μ_2_ are the viscosities of the pure ionic liquids, μ_m_ is the viscosity of the mixture, and *G* is a constant for a particular binary mixture. The *G* values for the ionic liquid mixture [C_4_^4^CNPy]_*x*_[C_4_C_1_Im]_1–*x*_[NTf_2_] are reported in the SI, but for 303 K, the value of *G* is −0.43. As the *G* value is negative, this indicates a lower than expected viscosity, which is more fluid in nature, indicating no strong associative interactions between the respective ions of the mixture. If the system behaved under the Arrhenius model, the value would be 0. Viscosity, along with density, are mainly controlled by their composition and their intrinsic interactions.^56^ The *G* value is in the similar range of other reported ionic liquids, the range tested by Fillion et al. being 1.50 to −0.78 for a large range of ionic liquid mixtures.^57^ Further reported in the work by Fillion et al. is that mixtures that share a common anion had *G* values near 0, which agrees with our results.

### Thermal Behavior

The thermogravimetric analysis of both ionic liquids shows an initial small mass loss (<5%) occurring below 200 °C. This is likely due to the evaporation of residual water and other volatiles from contact with air or from the synthesis.^58^ For both [C_4_^4^CNPy]­[NTf_2_] and [C_4_^3^CNPy]­[NTf_2_], decomposition of the ionic liquid was found to occur in a single step. The onset of mass loss starts at 240 °C for both [C_4_^4^CNPy]­[NTf_2_] and [C_4_^3^CNPy]­[NTf_2_], and extrapolated onset temperature of 342 °C for [C_4_^4^CNPy]­[NTf_2_] and 331 °C for [C_4_^3^CNPy]­[NTf_2_]. Hardarce et al. reports 350 °C for [C_4_^4^CNPy]­[NTf_2_] and 320 °C for [C_4_^3^CNPy]­[NTf_2_].^31^ Similar ionic liquids, such as 1-butylpyridinium bis­(trifluoromethylsulfonyl)­imide [C_4_Py]­[NTf_2_], begin degrading at 336 °C. The glass transition temperature of [C_4_^4^CNPy]­[NTf_2_] and [C_4_^3^CNPy]­[NTf_2_], determined using DSC, was found to be −54.57 and −55.94 °C, respectively. Domańska et al. found a glass transition temperature of −54.15 °C for [C_4_^4^CNPy]­[NTf_2_] in good agreement with our result.^49^ All of the DSC and TGA curves can be found in the SI.

### NMR Spectroscopy

One- and two-dimensional NMR spectroscopy was used to characterize the two ionic liquids, [C_4_^4^CNPy]­[NTf_2_] and [C_4_^3^CNPy]­[NTf_2_], in the presence and absence of ethylene and ethane. The results along with the proton shifts of ethane and ethylene in DMSO–*D*_6_ [C_4_^4^CNPy]­[NTf_2_] and [C_4_^3^CNPy]­[NTf_2_] are presented in Table 1.

**Table 1:** Proton Chemical Shifts (ppm) for Ethylene (=CH_3_) and Ethane (−CH_3_) in DMSO–*D*_6_,^59^ [C_4_^4^CNPy]­[NTf_2_], and [C_4_^3^CNPy]­[NTf_2_]

	ethane	ethylene
DMSO–*D*_6_	0.82	5.41
[C_4_^4^CNPy][NTf_2_]	0.74	5.29
[C_4_^3^CNPy][NTf_2_]	0.73	5.29

The proton chemical shifts for the gases are very similar in both ionic liquids and slightly upfield of the respective resonances in DMSO–*D*_6_. This indicates that the gases are in similar environments in both ionic liquids. NOESY NMR results from the [C_4_^4^CNPy]­[NTf_2_] and [C_4_^3^CNPy]­[NTf_2_] mixtures with the pure hydrocarbon gases show the expected intramolecular interactions, but no strong interaction with ethylene or ethane (figures in the SI) as expected for purely physisorbent ionic liquids with only weak van der Waals interactions between the solute and solvent.^60^

DOSY experiments confirmed this observation since there was either no difference or a small increase in the cation self-diffusion coefficient after the addition of both ethane and ethylene, as seen in Table 2 (and figures in the SI). In fact, the cation’s self-diffusion coefficients in ethane or ethylene presence in the different ionic liquids correlate well with the relative viscosity of the ionic liquids. We have made similar observations before, where physisorbed hydrocarbon gases had a diluting effect in the diffusion coefficients of cations of ionic liquids.^60^ If strong gas–ionic liquid interactions occurred, we could expect a competing effect between the dilution effect and a dragging effect from the attachment of ethylene to the ionic liquid cation, particularly in comparison with the effect of the noncoordinating ethane. Here, we only see evidence of a slight dilution effect of the gases in the IL, leading to an increase in their mobility.

**Table 2:** Self-Diffusion Coefficients, Represented in Different Forms, *D*_log_ in log­(m^2^ s^–1^) or *D* in 10^–12^(m^2^ s^–1^), of the Cation in the Ionic Liquids in the Absence (*D*) and Presence of Ethylene (*D*_ethylene_) and Ethane (*D*_ethane_)

ionic liquid	gas	*D*_log_	*D*_log,gas_	*D*	*D*_gas_
[C_4_^4^CNPy][NTf_2_]^a^	C_2_H_4_	–11.7	–11.6	2.2	2.5
	C_2_H_6_	–11.7	–11.7	2.0	2.0
[C_4_^3^CNPy][NTf_2_]^a^	C_2_H_4_	–11.8	–11.8	1.5	1.7
	C_2_H_6_	–11.8	–11.7	1.6	1.8
[C_4_C_1_Im][NTf_2_]^a^	C_2_H_4_	–10.6	–10.5	26.9	30.2
	C_2_H_6_	–10.6	–10.5	27.5	29.5

a The viscosity of the ionic liquid before bubbling of gas at 20 °C is 967.53 η/mPa·s for [C_4_^4^CNPy]­[NTf_2_], 1685.40 η/mPa·s for [C_4_^3^CNPy]­[NTf_2_], and 64.07 η/mPa·s for [C_4_C_1_Im]­[NTf_2_]. The water content of the ionic liquids is below <400 ppm.

### Mixing of Ionic Liquids

The excess molar volumes, *V* ^E^, were determined from the density values according to eq 16

16 $$V^{E}=\frac{\left(x_{\mathrm{IL}1}M_{\mathrm{IL}1}+x_{\mathrm{IL}2}M_{\mathrm{IL}2}\right)}{\rho_{\mathrm{mix}}}-\frac{\left(x_{\mathrm{IL}1}M_{\mathrm{IL}1}\right)}{\rho_{\mathrm{IL}1}}-\frac{\left(x_{\mathrm{IL}2}M_{\mathrm{IL}2}\right)}{\rho_{\mathrm{IL}2}}$$

where ρ_mix_ and ρ_IL_ are the densities of the mixture and the pure ionic liquids, respectively, *x*_IL_ is the mole fraction of each ionic liquid in the mixture, and *M*_IL_ is the molecular weight. For mixtures of [C_4_C_1_Im]­[NTf_2_] and [C_4_^4^CNPy]­[NTf_2_], the excess molar volumes are positive in the range of temperature investigated, and in the whole composition range, indicating a volumetric expansion of the systems as seen in Figure 5. The excess molar volume of the mixture [C_4_^4^CNPy]­[NTf_2_] and [C_4_C_1_Im]­[NTf_2_] indicates that the mixture expanded between 0 and 0.30 cm^3^ mol^–1^ at 20 °C.

**Figure 5:**
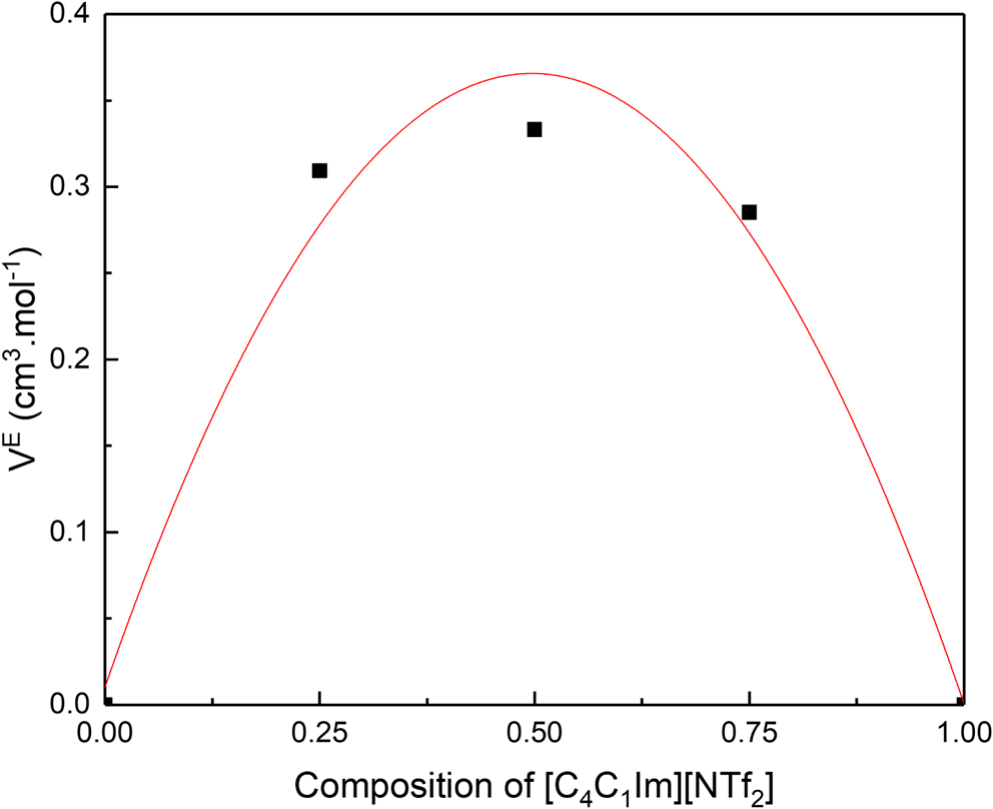
Excess molar volumes for the mixture of [C_4_^4^CNPy]­[NTf_2_] and [C_4_C_1_Im]­[NTf_2_] determined at 20 °C.

Otero et al. also investigated, using the same technique, mixtures of two ionic liquids with differing anions and cations, including [C_4_C_1_Im]^+^ combined with the bis­(trifluoromethylsulfonyl)­imide and acetate anions.^61^ The authors report a range of excess molar volumes for the 50:50 composition between 0 and 0.60 cm^3^ mol^–1^ similar to those obtained here. Furthermore, they found that larger positive excess molar volumes are found for mixtures containing ions of different sizes.^61^ Canongia Lopes et al. also studied binary mixtures of 1-methyl-*n*-alkylimidazolium bis­(trifluoromethylsulfonyl)­amide ionic liquids and reported small positive excess molar volume values, and closer to ideal mixtures as the differences in size between cation pairs were reduced in the order of a few tenths of cm^3^ mol^–1^. Therefore, the increased excess molar volume for our mixtures follows the trends previously observed.

The enthalpy of mixing of [C_4_C_1_Im]­[NTf_2_] and [C_4_^4^CNPy]­[NTf_2_] was calculated from the measured partial molar excess enthalpies. The values at several compositions were fitted by a Redlich–Kister equation and are shown in Figure 6. These ionic liquids mix endothermically with a maximum in the excess enthalpy being observed near the equimolar composition, +0.084 kJ mol^–1^.

**Figure 6:**
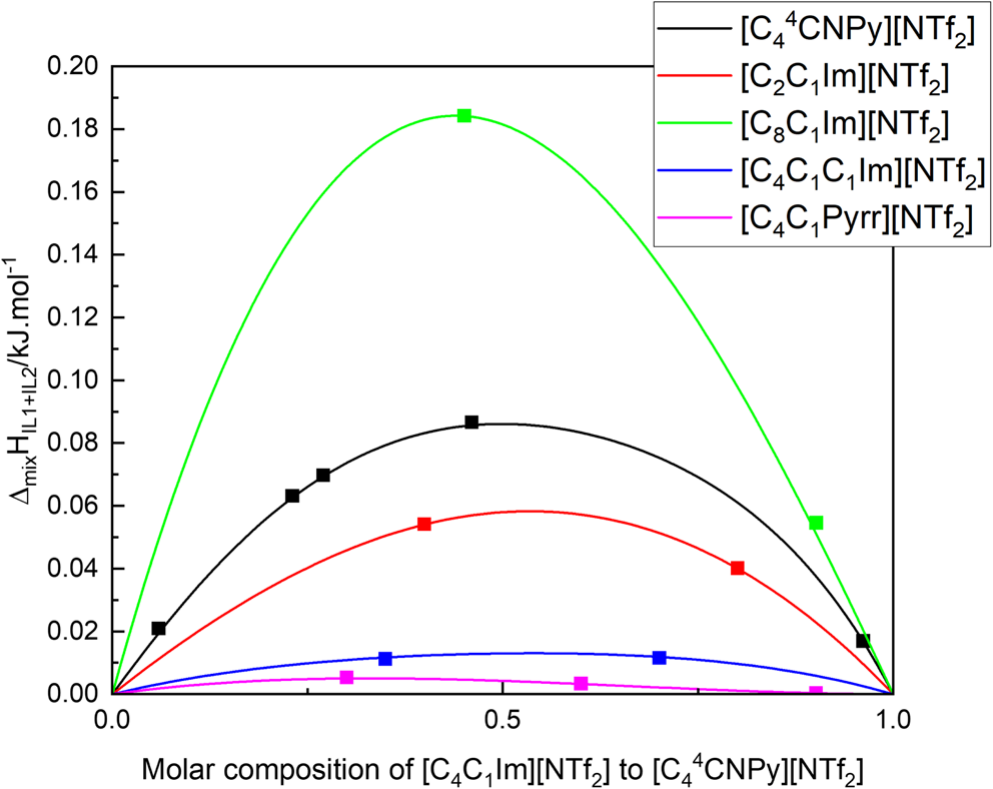
Molar enthalpies of mixing, Δ_mix_ *H*, of several IL1 + IL2 binary ionic liquid mixtures. The black line represents the molar enthalpy of mixing of the [C_4_^4^CNPy]­[NTf_2_] (IL1) + [C_4_C_1_Im]­[NTf_2_] (IL2) mixture studied in this work, with the black squares being the compositions at which the injections were performed. The experimental data can be found in the SI. Literature data includes [C_4_C_1_Im]­[NTf_2_] (IL1) mixed with several IL2:^62^ [C_2_C_1_Im]­[NTf_2_] (red), [C_8_C_1_Im]­[NTf_2_] (green), [C_4_C_1_C_1_Im]­[NTf_2_] (blue), and [C_4_C_1_Pyrr]­[NTf_2_] (magenta).

We compared the enthalpy of mixing measured herein with that of other ionic liquid mixtures. When [C_4_C_1_Im]­[NTf_2_] is mixed with [C_n_C_1_Im]­[NTf_2_], positive values were obtained varying from +0.058, +0.051, +0.182, and +0.359 kJ mol^–1^ for *n* = 2, 6, 8, and 10, respectively. This observation is consistent with the observed excess volumes for the same mixtures.^62,63^ Mixtures of [C_4_C_1_Im]­[NTf_2_] with [C_4_C_1_Pyrr]­[NTf_2_] (*N*-butyl-*N*-methylpyrrolidinium bis­(trifluoromethylsulfonyl)­imide) and of [C_4_C_1_Im]­[NTf_2_] with [C_1_C_1_C_4_Im]­[NTf_2_] show enthalpies of mixing close to the ideal mixture with small changes over composition—+ 0.004 and +0.013 kJ mol^–1^, respectively.^62^ The values of this work for the mixture of [C_4_C_1_Im]­[NTf_2_] and [C_4_^4^CNPy]­[NTf_2_] show a similar behavior with a small but positive enthalpy of mixing.

### Ethylene and Ethane Solubility Measurements

The experimental data obtained for the solubilities of ethylene and ethane in [C_4_^4^CNPy]­[NTf_2_], [C_4_^3^CNPy]­[NTf_2_], and [C_4_^4^CNPy]_0.5_[C_4_C_1_Im]_0.5_[NTf_2_] between 303 and 333 K and pressures up to 0.2 MPa is plotted in Figure 7, and data is reported in the SI.

**Figure 7:**
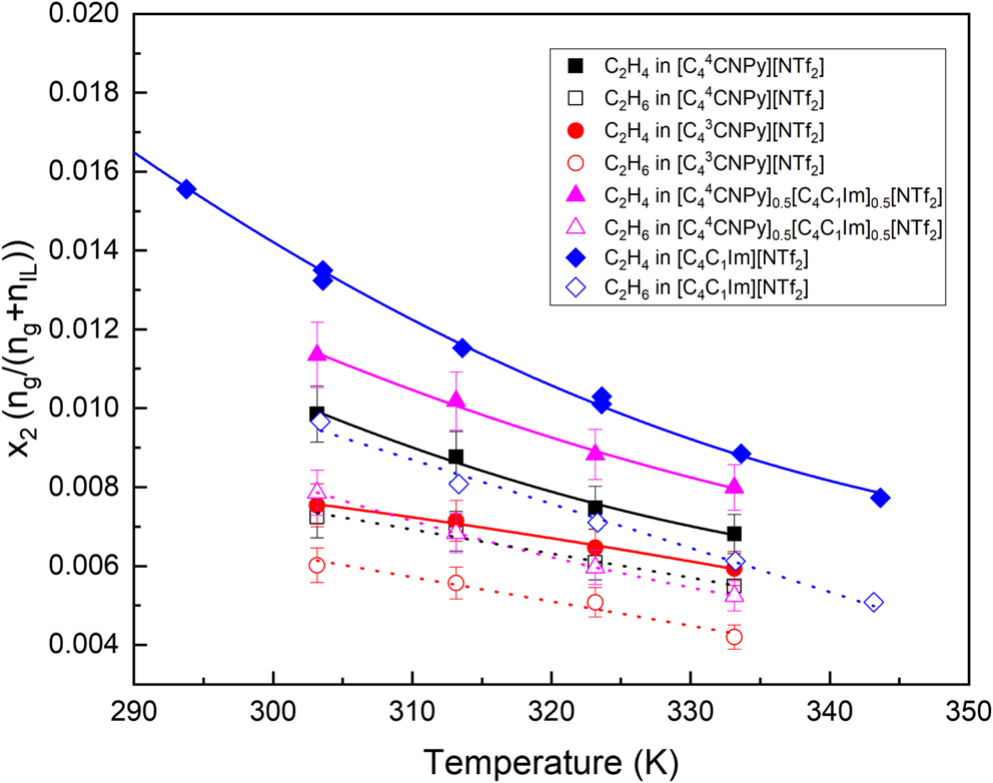
Mole fraction solubilites of ethylene (full symbols) and ethane (empty symbols) at 0.1 MPa and as a function of the temperature in the following ionic liquids: [C_4_^4^CNPy]­[NTf_2_] (square), [C_4_^3^CNPy]­[NTf_2_] (circle), [C_4_^4^CNPy]_0.5_[C_4_C_1_Im]_0.5_[NTf_2_] (triangle), and [C_4_C_1_Im]­[NTf_2_] (diamond) for comparison.^64,65^ The error bars are associated with the uncertainty in equipment and calculations.

The solubility of both gases in each of the ionic liquids decreases with temperature. Ethane and ethylene generally follow similar solubility trends in the ionic liquids: [C_4_C_1_Im]­[NTf_2_] > [C_4_^4^CNPy]_0.5_[C_4_C_1_Im]_0.5_[NTf_2_] > [C_4_^4^CNPy]­[NTf_2_] > [C_4_^3^CNPy]­[NTf_2_], from the highest to lowest solubility.

On average, ethylene solubility was higher than for its unsaturated counterpart in all ionic liquids tested at all temperatures, which is generally observed in low-molecular-weight physisorbent ionic liquids (Figure 8), and has been related to more favorable entropies of solvation for the unsaturated gas.^23^

**Figure 8:**
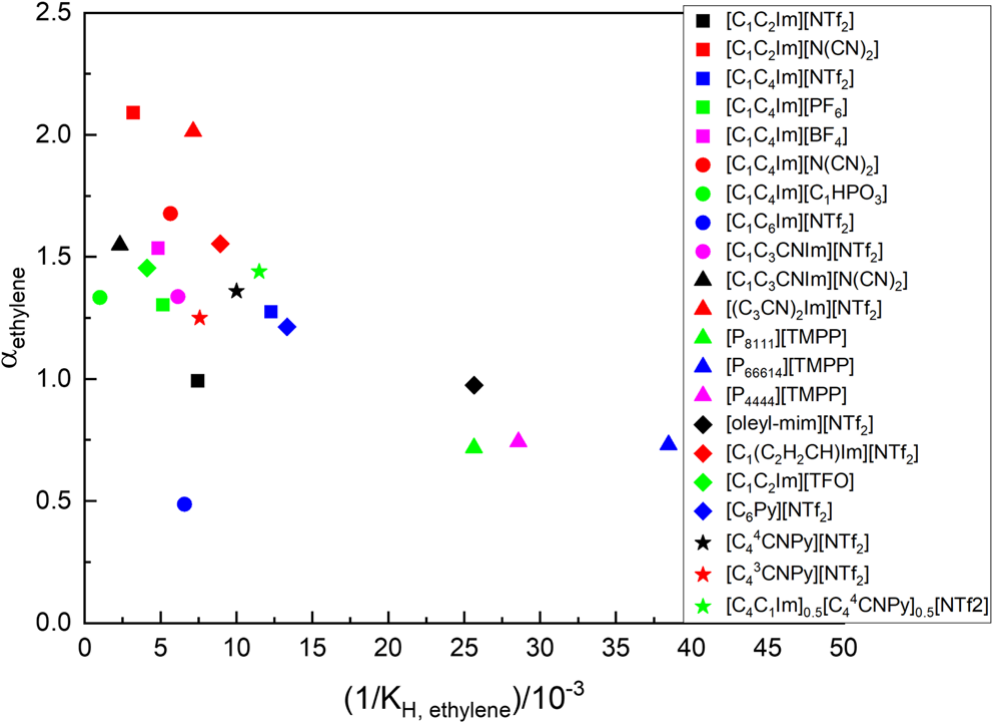
Ideal selectivity toward ethylene vs solubility at 313 K for reported literature data for ethylene and ethane separation. Literature work to date: 1-ethyl-3-methylimidazolium bis­(trifluoromethylsulfonyl)­imide [C_2_C_1_Im]­[NTf_2_] (■), 1-ethyl-3-methylimidazolium dicyanamide [C_1_C_2_Im]­[DCA] (red square), [C_4_C_1_Im]­[NTf_2_] (blue square), 1-butyl-3-methylimidazolium hexafluorophosphate [C_1_C_4_Im]­[PF_6_] (green square), 1-butyl-3-methylimidazolium tetrafluoroborate [C_1_C_4_Im]­[BF_4_] (magenta square), 1-butyl-3-methylimidazolium dicyanamide [C_4_C_1_Im]­[DCA] (red bullet), 1-butyl-3-methylimidazolium methylphosphite [C_1_C_4_Im]­[C_1_HPO_3_] (green bullet), 1-hexyl-3-methylimidazolium bis­(trifluoromethylsulfonyl)­imide [C_1_C_6_Im]­[NTf_2_] (blue bullet), 1-(3-cyanopropyl)-3-methylimidazolium bis­(trifluoromethylsulfonyl)­imide [C_1_C_3_CNIm]­[NTf_2_] (magenta bullet), 1-(3-cyanopropyl)-3-methylimidazolium dicyanamide [C_1_C_3_CNIm]­[DCA] (black triangle), 1,3-(3-cyanopropyl)­imidazolium bis­(trifluoromethylsulfonyl)­imide [(C_3_CN)_2_Im]­[NTf_2_] (red triangle), trimethyl-tetraoctylphosphonium bis­(2,4,4-trimethylpentyl) phosphinate [P_8111_]­[TMPP/DiOP] (green triangle), trihexyl-tetradecylphosphonium bis­(2,4,4-trimethylpentyl) phosphinate [P_66614_]­[TMPP/DiOP] (blue triangle), tetrabutylphosphonium bis­(2,4,4-trimethylpentyl) phosphinate [P_4444_]­[TMPP/DiOP] (magenta triangle), 1-(*Z*-octadec-9-enyl)-3-methylimidazolium bis­(trifluoromethylsulfonyl)­imide [oleyl-mim]­[NTf_2_] (black lozenge), 1-methyl-3-(propyn-3-yl)­imidazolium bis­(trifluoromethylsulfonyl)­imide [C_1_(C_2_H_2_CH)­Im]­[NTf_2_] (red lozenge), 1-hexylpyridinium bis­(trifluoromethylsulfonyl)­imide [C_6_Py]­[NTf_2_] (blue lozenge). From this work: ★ is [C_4_^4^CNPy]­[NTf_2_], red star is [C_4_^4^CNPy]­[NTf_2_], and green star is [C_4_^4^CNPy]_0.5_[C_4_C_1_Im]_0.5_[NTf_2_].

For the [C_4_^4^CNPy]_0.5_[C_4_C_1_Im]_0.5_[NTf_2_] mixture, gas solubilities closely follow the 50% weighted average of the gases’ solubility in pure ionic liquids. This is in agreement with results from the density, viscosity, and enthalpy of mixing that indicate the two ionic liquids mix ideally and behave independently of each other. In turn, the addition of [C_4_C_1_Im]­[NTf_2_] to [C_4_^4^CNPy]­[NTf_2_] does not promote stronger ethylene interactions with the latter.

Overall, these results indicate that both gases are simply physisorbed by the ionic liquids tested, with no indication of specific gas–liquid interactions occurring, meaning that solely van der Waals interactions are present.

The introduction of nitrile groups in the cation of the imidazolium or pyrolidinium ionic liquids seems to lead to a decrease in the solubility of both gases but also to a slight increase in the ethylene separation selectivity, as we can see by comparing the results for the pairs [C_4_C_1_Im]­[NTf_2_] and [C_1_C_3_CNIm]­[NTf_2_] (1-(3-cyanopropyl)-3-methylimidazolium bis­(trifluoromethylsulfonyl)­imide) and [C_6_Py]­[NTf_2_] and [C_4_^4^CNPy]­[NTf_2_] in Table 3. However, the decrease in solubility is smaller, and the increase in selectivity is higher for the pyrolidinium-based ionic liquids. Ethylene solubility is also more significantly affected by the small change of the CN functional group from position 3 to 4 on the cyanopyridinium cation, leading to a slight increase in its solubility.

**Table 3:** Mole Fraction Solubility of Ethylene and Ethane at 0.1 MPa and 313 K for the Ionic Liquids Used in This Work

ionic liquid	*x*_ethylene_	_ethane_	α_ethylene_	reference
[C_4_^4^CNPy][NTf_2_]	0.0099	0.0072	1.4	This work
[C_4_^4^CNPy][NTf_2_] (mixed gas)	0.0037	0.0036	1.0	This work
[C_4_^3^CNPy][NTf_2_]	0.0075	0.0060	1.3	This work
[C_4_^4^CNPy]_0.5_[C_4_C_1_Im]_0.5_[NTf_2_]	0.0113	0.0079	1.4	This work
[C_4_C_1_Im][NTf_2_]	0.0127	0.0087	1.5	Moura,^64^ Costa Gomes^65^
[C_6_Py][NTf_2_]	0.0133	0.0110	1.2	Anderson^66^
[C_1_C_3_CNIm][NTf_2_]	0.0066	0.0044	1.5	Moura^28^

[C_4_^4^CNPy]­[NTf_2_] was also used by Karpińska et al. for another saturated/unsaturated hydrocarbon separation, 1-hexene and hexane.^67,68^ In this case, the selectivity toward the unsaturated hydrocarbon was between 1.5 and 2.5. This value is in a similar range to those obtained for other ionic liquids for the 1-hexene/hexane separation, such as [C_4_C_1_Im]­[DCA], confirming the existence of only weak interactions with all these ionic liquids.^67,68^

To complete our study, we determined the mixed gas solubility of a 50:50 molar mixture of ethylene and ethane in [C_4_^4^CNPy]­[NTf_2_] at 30 °C to mimic more realistic separation conditions. [C_4_C_1_Im]­[NTf_2_] was chosen as the ionic liquid has been extensively studied and characterized with a relatively low viscosity. The matching anion will prevent any external influence on the mixing with the cyanopyridinium ionic liquid. For pure physisorbent materials and at these low solubility values, we would not expect that the solubility of one gas should have a large impact on the other; however, ethylene selectivity decreased from 1.4 to 1.0 (as seen in Table 3). This could be due to the similarities in the absorption mechanisms for both gases, and competition for the same solvation sites. This will be justified when investigating the radial distribution functions (Figure 10), where the most probable positions of ethylene and ethane relative to different atoms on the cation overlap with each other.

The calculated values for the thermodynamic properties of solvation of the studied gases in [C_4_^4^CNPy]­[NTf_2_], [C_4_^3^CNPy]­[NTf_2_], and [C_4_^4^CNPy]_0.5_[C_4_C_1_Im]_0.5_[NTf_2_] at 313 K are plotted in Figure 9 alongside with reported data for [C_4_C_1_Im]­[NTf_2_].^64,65^ The remainder of the data can be found in the SI. Solvation of both gases in all the ionic liquids tested is ruled by the entropic term, which plays an unfavorable contribution to the process. Similar trends have been seen for other ionic liquids, notably for the pair [C_4_C_1_Im]­[NTf_2_]/[C_1_C_3_CNIm]­[NTf_2_] in that the introduction of a cyano group in the cation of the ionic liquids let to a decrease in the solubility of both gases due to a less favorable entropic term.^28^

**Figure 9:**
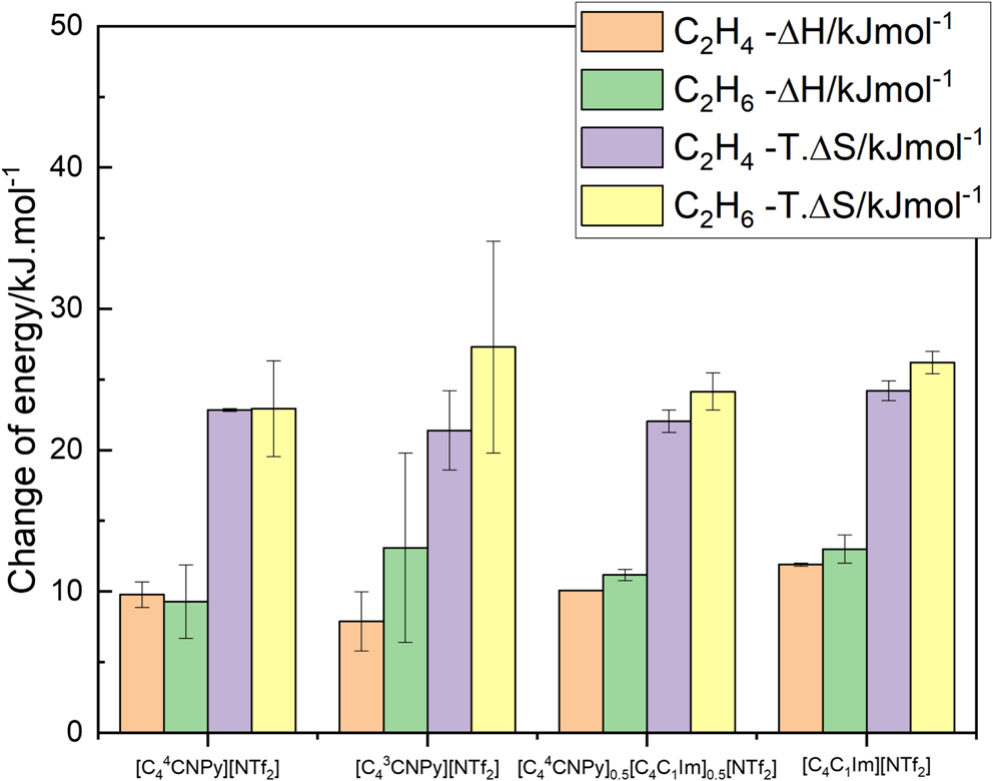
Graphical representation of the enthalpic and entropic contributions to the solubility of the studied gases in the cyanopyridinium ionic liquids and the mixture of ionic liquids, at 0.1 MPa partial pressure and 323 K. Reported [C_4_C_1_Im]­[NTf_2_] data has been included as a reference.^64,65^ The error associated in the figure is due to the variation of the temperature.

Although Stassen et al. have suggested that interactions in ionic liquids might explain entropy-driven solvation,^69^ we found no clear evidence of gas–ionic liquid interactions as the enthalpy of solvation of the gases studied was low and similar in all the studied pairs.^69^ However, the uncertainty associated with the thermodynamic properties of solvation (that derive from variation in the temperature of measurements) makes it difficult to clearly distinguish between the role of the enthalpic and entropic terms in the preferential solubility of ethylene or ethane in the ionic liquids.

### MD Simulations

Molecular dynamics (MD) simulations of the [C_4_C_1_Im]­[NTf_2_], [C_4_^3^CNPy]­[NTf_2_], and [C_4_^4^CNPy]­[NTf_2_] ionic liquids in their pure forms and containing an ethane or an ethylene molecule were performed to examine solute solvation. Figure 10 shows the radial distribution functions (RDFs) between C/N atoms on the cations and the C atoms of the two gas molecules, for the [C_4_C_1_Im]­[NTf_2_], [C_4_^4^CNPy]­[NTf_2_], and [C_4_^3^CNPy]­[NTf_2_] ionic liquids, respectively. The results of [C_4_C_1_Im]­[NTf_2_] of Figure 10 are in agreement with previous MD simulations reported in the literature, which suggest that the most probable location for both ethylene and ethane in [C_4_C_1_Im]­[NTf_2_] is close to the terminal (C8 on the alkyl chain in Figure 10) carbon on the alkyl chain.^70^ The agreement of the RDFs for this cation with those previously reported gives us confidence in the ability of the potentials for [C_4_^4^CNPy]­[NTf_2_] and [C_4_^3^CNPy]­[NTf_2_] to model cation–gas interactions.^70^

**Figure 10:**
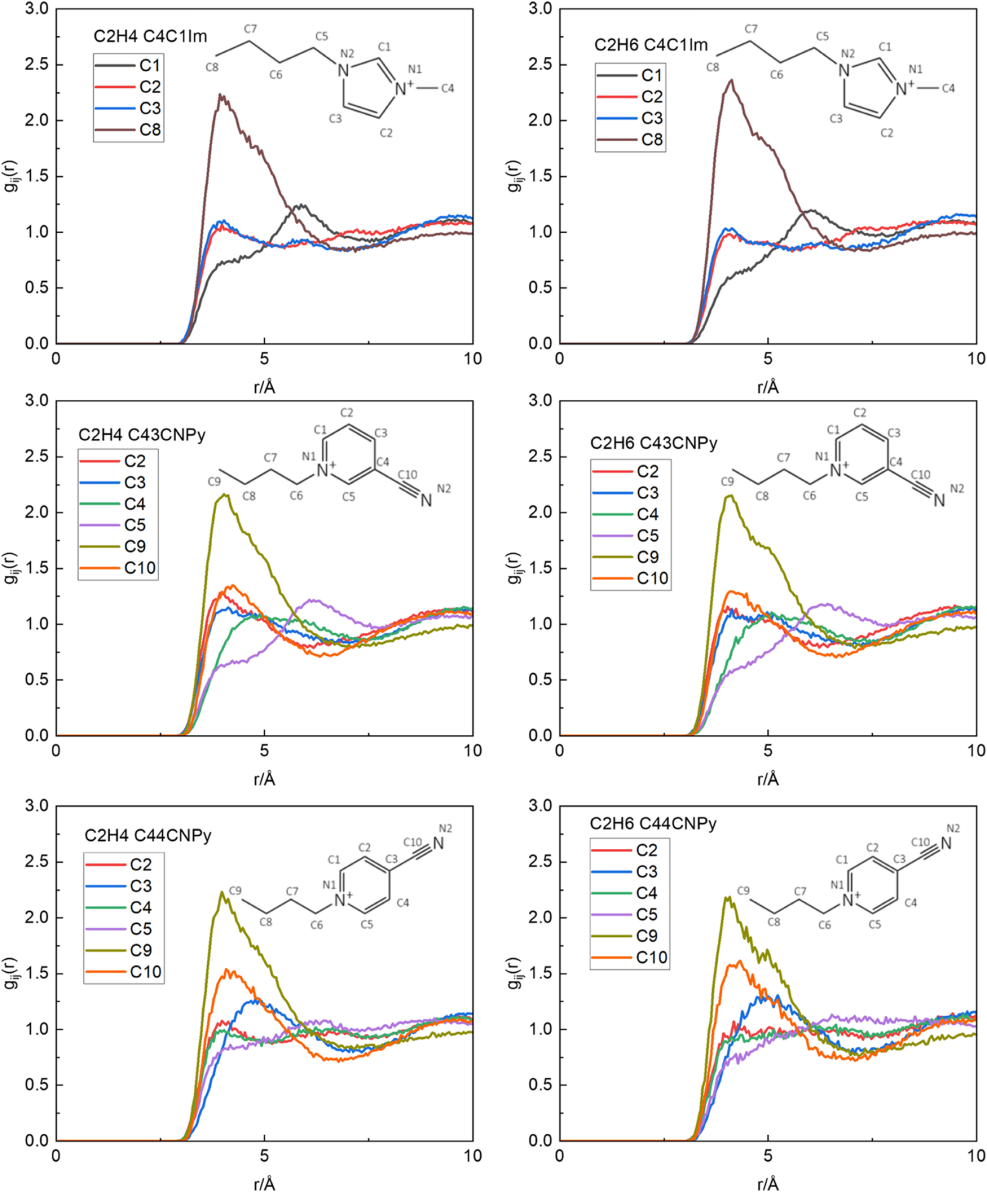
Radial distribution function for pairs of atoms for the carbon atom of ethylene (left column) and ethane (right column) for a cation of [C_4_C_1_Im]­[NTf_2_] (top), [C_4_^3^CNPy]­[NTf_2_] (middle), and [C_4_^4^CNPy]­[NTf_2_] (bottom).

As can be seen in Figure 10 for [C_4_^4^CNPy]­[NTf_2_] and [C_4_^3^CNPy]­[NTf_2_], there are no large differences in the RDFs for association of ethylene and ethane with either ionic liquid cation. This supports the experimental observations that the interaction environments of the two gas molecules are similar and that in mixed gas studies, competition may play a significant role. Our simulation results confirm the experimental observations, and no significant differences between the structuring of the gas molecules around the two cations were noted, directly confirming from an energetic point of view that ethylene does not interact more strongly with the cation than does ethane. There are subtle, though statistically significant, differences between the RDFs, which are shown in more detail in the SI: This suggests that MD simulations are able to qualitatively capture the slight differences observed experimentally. Preliminary free-energy calculations did not give a statistically significant difference between the free energy of solvation of the two molecules in any of the ionic liquids, indicating that impractically large simulation times will be needed to converge these quantities.

A closer viewpoint on these subtle differences is given by the density plots of Figure 11, which show the local density of gas molecules projected onto the aromatic ring in each ionic liquid’s cation, as described in the SI. Although the local structure of the gases around the ring differs in shape, the magnitudes of these differences are quite small, although statistically significant. This is in accordance with our experimental conclusions that there is no strong preference for ethylene over ethane.

**Figure 11:**
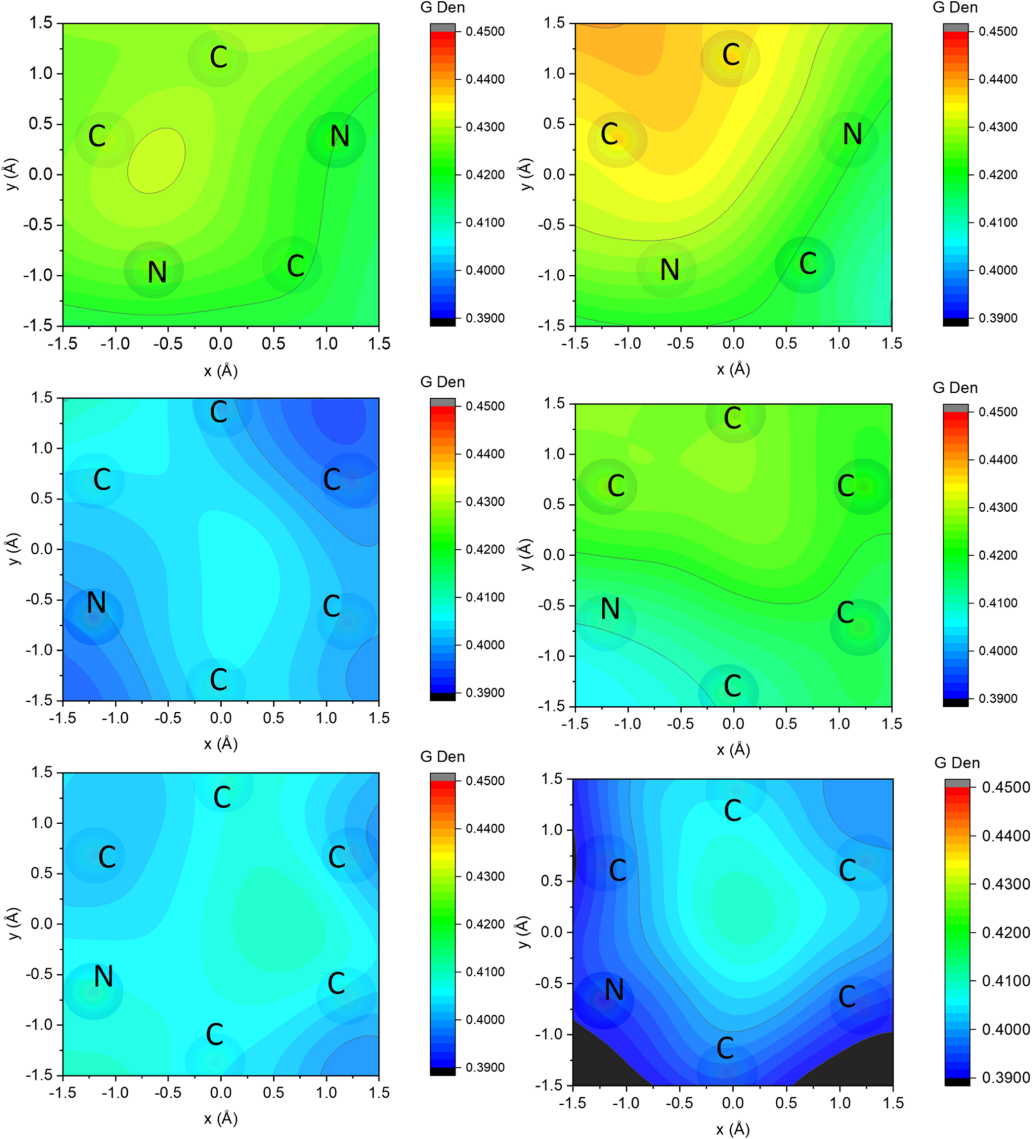
Six histograms show the local density of gas molecules projected onto the aromatic ring in each ionic liquid’s cation. Top left is ethylene onto [C_4_C_1_Im]­[NTf_2_], and top right is ethane onto [C_4_C_1_Im]­[NTf_2_]. Middle left is ethylene onto [C_4_^3^CNPy]­[NTf_2_], and middle right is ethane onto [C_4_^3^CNPy]­[NTf_2_]. Bottom left is ethylene onto [C_4_^4^CNPy]­[NTf_2_], and bottom right is ethane onto [C_4_^4^CNPy]­[NTf_2_].

Comparing differences between the gas-cation RDFs for the two isomeric cyanopyridinium cations, the largest correlation to the gas molecules in both cases is with the terminal methyl site on the butyl chain. This is also consistent with the simulation with the [C_4_C_1_Im]^+^ cation in Moura.^70^ In terms of solvation by the cyanopyridinium ring sites from Figure 10, the 4-cyano isomer shows a higher degree of association in the RDF associated with ethylene and ethane to nitrile interactions compared with that in the 3-cyano isomer. This may be a function of the differences in geometries of these two isomeric cations, but may also reflect subtle differences in the modes of solvation that reflect the increased solubilities of both gases in the 4-cyano ionic liquid.

## Conclusions

In this paper, we examined whether the separation of ethylene and ethane through physisorption in ionic liquids could be enhanced by the use of cyanopyridinium ionic liquids, through association with the cationic cyanopyridinium ring of the ionic liquids. This would emulate the strong interaction found between these ionic liquids and polymaromatic molecules, which have been found to form charge-transfer complexes.^31^ The solubility of ethylene and ethane in two ionic liquids: [C_4_^4^CNPy]­[NTf_2_] and [C_4_^3^CNPy]­[NTf_2_] was determined, as was the ethylene separation ideal selectivity. The values for both ionic liquids were found to be similar to those previously reported for other ionic liquids of similar molecular weight (Figure 8), where no specific gas–ionic liquid interactions are formed. To eliminate the possibility of steric constrictions in the formation of charge-transfer complexes with ethylene, [C_4_C_1_Im]­[NTf_2_] was added as a diluent, with the added benefit of obtaining an absorbent with lower viscosity. The mixture containing [C_4_^4^CNPy]­[NTf_2_] and [C_4_C_1_Im]­[NTf_2_] was found to mix in an almost ideal manner with gas solubilities presenting as a weight of those of the two pure ionic liquids. NMR spectroscopy and molecular dynamic simulations were used to obtain more information on our systems at the molecular level and both showed that no dominant interaction occurred between the gas and the ionic liquids. Taken together, our results confirm that the ethane and ethylene solubilities in these cyanopyridinium ionic liquids are ruled by nonspecific gas–ionic liquid interactions, and that the nitrile group does not have any specific additional benefit. The results point toward solvation taking place preferentially in the nonpolar domains of the ionic liquids.

## Supplementary Material



## Notes

Ionic liquids’ synthesis methodology, ^1^H NMR and ^13^C NMR characterization of ionic liquids and intermediates, CHNS and mass spectrometry analysis of ionic liquids, experimental density measurements, linear fits of density with temperature, experimental viscosity measurements, Vogel–Fulcher–Tammann (VFT) equation fits for viscosity, viscosity data and VFT fits plots, data on effect of temperature on the value of *G* for the ionic liquid mixture, TGA and DSC methodology and data, mass spectrometry method, 2D NMR method and DOSY and NOESY data, gas solubility system benchmarking and calibration information and data, gas solubility data, plots and fits, thermodynamic functions of solvation of the gases in the ionic liquids, calorimetry of ionic liquid mixing data and plot; MD simulation and quantum-mechanical calculations along with brief descriptions (PDF)
