# Supplementary material for: Cyanopyridinium-Based Ionic Liquids and Their Mixtures for Ethylene and Ethane Separation
Source: ACS Sustain Chem Eng. 2025 Jul 24;13(30):11770–83. doi: 10.1021/acssuschemeng.5c01481 (PMC12326391; doi:10.1021/acssuschemeng.5c01481)
Supplement: Supplementary file 1 [file sc5c01481_si_001.pdf]

Cyanopyridinium-based ionic liquids and their mixtures for ethylene and ethane separation

Sam H. McCalmont,[†] Guillaume Simon,[‡] H. Q. Nimal Gunaratne,[¶] Margarida Costa Gomes,[‡] David M. Wilkins,[§] John D. Holbrey,[¶] and Leila Moura[*,¶]

†*QUILL Research Centre, School of Chemistry and Chemical Engineering, Queen's University Belfast, Belfast BT9 5AG, Northern Ireland, UK*

‡*Laboratoire de Chimie de l'ENS Lyon, CNRS and Université de Lyon, 46 allée d'Italie, 69364 Lyon, France*

¶*QUILL Research Centre, School of Chemistry and Chemical Engineering, Queen's University Belfast, BT9 5AG, Northern Ireland, UK*

§*Centre for Quantum Materials and Technologies, School of Mathematics and Physics, Queen's University Belfast, Belfast BT7 1NN, Northern Ireland, UK*

E-mail: l.moura@qub.ac.uk

Supplementary Information

Contents

1 Synthesis **S7**

1.1 Synthesis of N-butyl-4-cyanopyridinium bromide, $[C_4^4CNPy]Br$ S7

1.2 Synthesis of N-butyl-4-cyanopyridinium bis(trifluoromethanesulfonyl)imide, $[C_4^4CNPy][NTf_2]$ S7

1.3 Synthesis of N-butyl-3-cyanopyridinium bromide, $[C_4^3CNPy]Br$ S8

1.4 Synthesis of N-butyl-3-cyanopyridinium bis(trifluoromethanesulfonyl)imide, $[C_4^3CNPy][NTf_2]$ S9

1.5 Synthesis of 1-butyl-3-methylimidazolium chloride, $[C_4C_1Im]Cl$ S10

1.6 Synthesis of 1-butyl-3-methylimidazolium bis(trifluoromethanesulfonyl)imide, $[C_4C_1Im][NTf_2]$ S10

2 Characterisation **S11**

2.1 Density and viscosity S11

2.2 Thermal stability S15

2.3 Phase behaviour S17

2.4 Mass spectrometry S18

3 2D NMR **S18**

4 Gas solubility **S24**

4.1 Gas solubility system (GSS) S24

4.2 Pure gas solubility data S27

4.3 Mixed gas solubility data S31

4.4 Calorimetry S34

List of Figures

S1 Experimental viscosities (η/mPa · s) of the ionic liquids studied against temperature (K), the lines on the experimental data represent the fitting of the VFT parameters as seen in the manuscript. S13

S2 TGA of [C_4^4CNPy][NTf_2] S15

S3 TGA of [C_4^3CNPy][NTf_2] S16

S4 DSC of [C_4^4CNPy][NTf_2] (exothermic down) S18

S5 DSC of [C_4^3CNPy][NTf_2] (exothermic down) S19

S6 DOSY NMR of [C_4^3CNPy][NTf_2] (left) and [C_4^3CNPy][NTf_2] (right) containing ethane. The ethane peak is highlighted by the circles. S20

S7 DOSY NMR of [C_4^3CNPy][NTf_2] (left) and [C_4^3CNPy][NTf_2] (right) containing ethylene. The ethylene peak is highlighted by the circles. S20

S8 NOESY NMR of [C_4^3CNPy][NTf_2] (left) and [C_4^3CNPy][NTf_2] (right) containing ethane. The ethane peak is highlighted by the circles. S21

S9 NOESY NMR of [C_4^3CNPy][NTf_2] (left) and [C_4^3CNPy][NTf_2] (right) containing ethylene. The ethylene peak is highlighted by the circles. S21

S10 DOSY NMR of [C_4^4CNPy][NTf_2] (left) and [C_4^4CNPy][NTf_2] (right) containing ethane. The ethane peak is highlighted by the circles. S22

S11 DOSY NMR of [C_4^4CNPy][NTf_2] (left) and [C_4^4CNPy][NTf_2] (right) containing ethylene. The ethylene peak is highlighted by the circles. S22

S12 NOESY NMR of [C_4^4CNPy][NTf_2] (left) and [C_4^4CNPy][NTf_2] (right) containing ethane. The ethane peak is highlighted by the circles. S23

S13 NOESY NMR of [C_4^4CNPy][NTf_2] (left) and [C_4^4CNPy][NTf_2] (right) containing ethylene. The ethylene peak is highlighted by the circles. S23
S14 Mole fraction of C_2H_4 in [C_4C_1Im][NTf_2] as a function of equilibrated pressure at 30 °C for this work. S26
S15 The partial molar excess enthalpies of [C_4C_1Im][NTf_2] (red line) and [C_4^4CNPy][NTf_2] (black line). S34

List of Tables

S1 CHNS analysis for $[C_4^4CNPy][NTf_2]$ S8

S2 CHNS analysis for $[C_4^3CNPy][NTf_2]$ S9

S3 Experimental values for densities (ρ/ g cm^{-3}) for the ionic liquids with the same anion ($[NTf_2]^-$) but differing cations to study the impact of the cyanopyridnium cation. S12

S4 Experimental values for viscosity (η/mPa$\cdot$s) for the ionic liquids with the same anion ($[NTf_2]^-$) S12

S5 Vogel-Fulcher-Tammann equation parameters. S13

S6 Effect of temperature on the value of G for the mixture of $[C_4C_1Im][NTf_2]$ + $[C_4^4CNPy][NTf_2]$ from 293.15 K to 303.15 K. S14

S7 TGA method used utilised for $[C_4^3CNPy][NTf_2]$ and $[C_4^3CNPy][NTf_2]$ S15

S8 DSC method used for both ionic liquids S17

S9 Raw experimental values for the solubility of ethylene in $[C_4C_1Im][NTf_2]$ on the GSS. S24

S10 The mole fraction of C_2H_4 in $[C_4C_1Im][NTf_2]$ using the GSSB. S25

S11 Experimental data for the solubility of ethylene in $[C_4^4CNPy][NTf_2]$. S27

S12 Experimental data for the solubility of ethane in $[C_4^4CNPy][NTf_2]$. S28

S13 Experimental data for the solubility of ethylene in $[C_4^3CNPy][NTf_2]$. S28

S14 Experimental data for the solubility of ethane in $[C_4^3CNPy][NTf_2]$. S29

S15 Experimental data for the solubility of ethylene in $[C_4^4CNPy]_{0.5}[C_4C_1Im]_{0.5}[NTf_2]$. S29

S16 Experimental data for the solubility of ethane in $[C_4^4CNPy]_{0.5}[C_4C_1Im]_{0.5}[NTf_2]$. . S30

S17 Parameters A_i used to fit the experimental results as a function of the Henry's Law constants, K_H as well as, the average relative deviation of the experimental points to the fit. S30

S18 Raw experimental values for the solubility of ethylene and ethane (as a 50:50 % molar mixture) in $[C_4^4CNPy][NTf_2]$. S31
S19 Thermodynamic functions of solvation of the gases studied in $[C_4^4CNPy][NTf_2]$ at several temperatures between 303 and 333 K. S32
S20 Thermodynamic functions of solvation of the gases studied in $[C_4^3CNPy][NTf_2]$ at several temperatures between 303 and 333 K. S32
S21 Thermodynamic functions of solvation of the gases studied in $[C_4^4CNPy]_{0.5}[C_4C_1Im]_{0.5}[NTf_2]$ at several temperatures between 303 and 333 K. S33
S22 Stoichiometric data and heat effects of the calorimetry experiments with the system $[C_4^4CNPy][NTf_2]$ (1) + $[C_4C_1Im][NTf_2]$ (2) at 303.15 K. S35

1 Synthesis

1.1 Synthesis of N-butyl-4-cyanopyridinium bromide, $[C_4^4CNPy]Br$

The original synthesis as proposed by Hardacre *et al.* stipulated the combination of 4-cyanopyridine and 1-bromobutane in a round bottom flask sealed with a Safe-Lab(R) pressure seal at 135 °C.[1] The temperature is required for the alkylation of 4-cyanopyridine. The experimental method was modified in favour of a reflux based apparatus. 4-Cyanopyridine (15.61 g, 150 mmol) and 1-bromobutane (30.81 g, 225 mmol)(1.5 molar equivalent) were added to 50 cm^3 of toluene. This was heated to 110 °C for 48 hours under reflux. The resultant yellow solid mass was filtered rapidly to remove excess 1-bromobutane and washed with toluene. The solid was then dried to leave a pale cream-coloured powder. The yield was 22 g (61%).

^{1}H-NMR (400 MHz, DMSO$-D_6$) δ (ppm): 9.42(2H, ds, $-C\mathbf{H}-CH-N$), 8.72 (2H, ds, $-CH-C\mathbf{H}-N$), 4.69 (2H, m, $-N-C\mathbf{H}_2-CH_3$), 1.91 (2H,m,$-CH_2-C\mathbf{H}_2-CH_3$), 1.29 (2H, m, $-C\mathbf{H}_2-CH_3$), 0.89 (3H, t, $-C\mathbf{H}_3$). ^{13}C-NMR (100 MHz, DMSO$-D_6$) δ (ppm): 145.6 (s, $N-\mathbf{C}H-CH-C$), 128.0 (s, $N-CH-\mathbf{C}H-C$), 115.1 (s, $\mathbf{C}N$), 115.8 (s, $\mathbf{C}-CN$), 62.0 (s, $N-\mathbf{C}H_2-CH_2-CH_3$), 33.1 (s, $N-CH_2-\mathbf{C}_2$), 19.2 (s,$-CH_2-\mathbf{C}H_2-CH_3$), 13.0 (s, $-CH_2-\mathbf{C}H_3$).

1.2 Synthesis of N-butyl-4-cyanopyridinium bis(trifluoromethanesulfonyl)imide, $[C_4^4CNPy][NTf_2]$

The metathesis of a 50 cm^3 solution of $[C_4^4CNPy]Br$ (22.05 g, 96 mmol) in water with 50 cm^3 of an aqueous solution of $Li[NTf_2]$ (28.05 g, 98 mmol)(1 mole equivalent) was left to mix for 24 hours. Two layers formed, the lower ionic liquid phase was separated and extracted into dichloromethane. The lower phase was collected and washed repeatedly with water until no bromide traces remained in the washings (tested with silver nitrate solution). Then the ionic liquid dried under reduced pressure with heat to produce a yellow-orange liquid (yield, 30 g, 71 %).

^{1}H-NMR (400 MHz, DMSO$-D_6$) δ (ppm): 9.37 (2H, ds, $-$C**H**$-$CH$-$N), 8.71 (2H, ds, $-$CH$-$C**H**$-$N), 4.66 (2H, m, $-$N$-$C**H**$_2-$CH$_3$), 1.91 (2H,m,$-$CH$_2-$C**H**$_2-$CH$_3$), 1.31 (2H, m, $-$C**H**$_2-$CH$_3$), 0.91 (3H,t,$-$C**H**$_3$). ^{13}C-NMR (100 MHz, DMSO$-D_6$) δ (ppm): 146.18 (s, N$-$**C**H$-$CH$-$C), 131.04 (s, N$-$CH$-$**C**H$-$C), 121.10 (s, **C**N), 117.90 (s, $-$**C**F$_3$), 114.84 (s, **C**$-$CN), 61.51 (s, N$-$**C**H$_2-$CH$_2-$CH$_3$), 33.29 (s, N$-$CH$_2-$**C**$_2$), 19.15 (s,$-$CH$_2-$**C**H$_2-$CH$_3$), 13.81 (s, $-$CH$_2-$**C**H$_3$).

Table S1: CHNS analysis for [C_4^4CNPy][NTf_2]

Molecule	Formula	Atoms (CHNS)	Predicted %	Found %
[C_4^4CNPy][NTf_2]	$C_{12}H_{13}N_3S_2O_4F_6$	C	32.66	32.82
		H	2.97	3.13
		N	9.52	9.39
		S	14.53	14.02

For [C_4^4CNPy][NTf_2] ([$C_{12}H_{13}O_4N_3F_6S_2$]), MS: +ve mode: 161 ([cation]$^+$ calc, 161); -ve mode 280 ([anion]$^-$ calc, 280) (The mass spec procedure is provided below).

1.3 Synthesis of N-butyl-3-cyanopyridinium bromide, [C_4^3CNPy]Br

The original synthesis as proposed by Hardacre *et al.* stipulated the combination of 3-cyanopyridine and 1-bromobutane in a round bottom flask sealed with a Safe-Lab(R) pressure seal at 135 °C. The experimental method was modified in favour of a reflux based apparatus. 3-Cyanopyridine (15.01 g, 144 mmol) and 1-bromobutane (29.61 g, 216 mmol)(1.5 molar equivalent) were added to 50 cm^3 of toluene. This was heated to 110 °C for 48 hours under reflux. The resultant yellow solid mass was filtered rapidly to remove excess 1-bromobutane and washed with toluene. The solid was then dried to leave a pale cream-coloured powder (yield, 19 g, 59 %).

^{1}H-NMR (400 MHz, DMSO$-D_6$) δ (ppm): 9.91 (1H, d, N$-$C**H**$-$C(CN)), 9.46 (1H, d, N$-$CH$-$CH$-$C**H**$-$C(CN)), 9.13 (1H, d, N$-$C**H**$-$CH$-$CH$-$C(CN)), 8.39 (1H, d, N$-$CH$-$C**H**$-$CH$-$C(CN)), 4.71 (2H, m, $-$N$-$C**H**$_2-$CH$_3$), 1.95 (2H ,m, $-$CH$_2-$C**H**$_2-$CH$_3$), 1.31 (2H, m, $-$C**H**$_2-$CH$_3$), 0.92 (3H, t, $-$C**H**$_3$). ^{13}C-NMR (100 MHz, DMSO$-D_6$) δ

(ppm): 149.9 (s, N−**C**H−CH), 148.8 (s, N−**C**H−C(CN), 128.9 (s, **C**H−**C**H−C(CN)), 114.3 (s, **C**N), 112.5 (s, **C**−CN), 61.5 (s, N−**C**H_2−CH_2−CH_3), 33.3 (s, N−CH_2−$\mathbf{C}_2$), 19.2 (s,−CH_2−**C**H_2−CH_3), 13.8 (s, −CH_2−**C**H_3).

1.4 Synthesis of N-butyl-3-cyanopyridinium bis(trifluoromethanesulfonyl)imide, [C_4^3CNPy][NTf_2]

The metathesis of a 50 cm^3 solution of [C_4^3CNPy]Br (12.26 g, 54 mmol) in water with 50 cm^3 of an aqueous solution of Li[NTf_2] (15.38 g, 54 mmol, equimolar) was left to mix for 24 hours. Two layers formed, the lower ionic liquid phase was separated and extracted into dichloromethane. The lower phase was collected and washed repeatedly with water until no bromide traces remained in the washings (tested with silver nitrate solution). Then the ionic liquid was dried under reduced pressure at 40 °C to produce a yellow-orange liquid. The yield was 16 g (71 %).

^{1}H-NMR (400 MHz, DMSO−D_6) δ (ppm): 9.80 (1H, d, N−C**H**−C(CN)), 9.33 (1H, d, N−CH−CH−C**H**−C(CN)), 9.10 (1H, d, N−C**H**−CH−CH−C(CN)), 8.36 (1H, d, N−CH−C**H**−CH−C(CN)), 4.61 (2H, m, −N−C**H**$_2$−CH_3), 1.93 (2H,m,−CH_2−C**H**$_2$−CH_3), 1.32 (2H, m, −C**H**$_2$−CH_3), 0.92 (3H,t,−C**H**$_3$). ^{13}C-NMR (100 MHz, DMSO−D_6) δ (ppm): 149.9 (s, N−**C**H−CH), 148.8 (s, N−**C**H−C(CN), 128.9 (d, **C**H−**C**H−C(CN)), 118.3 (s, −**C**F_3), 114.2 (s, **C**N), 113.5 (s, **C**−CN), 61.4 (s, N−**C**H_2−CH_2−CH_3), 32.7 (s, N−CH_2−$\mathbf{C}_2$), 18.5 (s,−CH_2−**C**H_2−CH_3), 13.7 (s, −CH_2−**C**H_3).

Table S2: CHNS analysis for [C_4^3CNPy][NTf_2]

Molecule	Formula	Atoms (CHNS)	Predicted %	Found %
[C_4^3CNPy][NTf_2]	$C_{12}H_{13}N_3S_2O_4F_6$	C	33.66	32.66
		H	2.97	3.10
		N	9.52	9.44
		S	14.53	14.16

For [C_4^3CNPy][NTf_2] ([$C_{12}H_{13}O_4N_3F_6S_2$]) MS: +ve mode: 161 ([cation]$^+$ calc, 161); -ve

mode 281 ([anion]$^-$ calc, 280) The mass spectrometry procedure is provided below.

1.5 Synthesis of 1-butyl-3-methylimidazolium chloride, $[C_4C_1Im]Cl$

1-Chlorobutane (128.79 g, 1391 mmol) was added to freshly distilled 1-methylimidazole (70.50 g, 859 mmol) dissolved in acetonitrile. The mixture was stirred for 48 hours at 65 °C. The mixture was concentrated by rotary evaporation, and dried overnight. An oily liquid was obtained, which crystallises into a white solid. The yield was 89 g (81 %).

^{1}H-NMR (400 MHz, DMSO$-D_6$) δ (ppm): 9.10 (1H, s, N$-$**CH**$-$N), 7.74 (2H, m, N$-$C**H**$-$C**H**$-$N), 4.16 (2H, t, N$-$C**H**$_2-CH_3$), 3.85 (3H, s,C**H**$_3-$N), 1.77 (2H, m,CH_2-C**H**$_2-CH_2-CH_3$), 1.28 (2H, m, CH_2-C**H**$_2-CH_3$), 0.90 (3H, t, CH_2-C**H**$_3$). ^{13}C-NMR (100 MHz, DMSO$-D_6$) δ (ppm): 135.7 (s, N$-$**C**H$-$N), 123.6 (s, N$-$**C**H$-$CH$-$N), 121.2 (s,N$-$CH$-$**C**H$-$N), 48.3 (s, N$-$**C**$_2$), 35.7 (s,N$-$**C**H$_3$), 31.3 (s, N$-CH_2-$**C**$_2$), 18.7 (s,$-CH_2-$**C**H$_2-CH_3$), 13.3 (s, $-CH_2-$**C**H$_3$).

1.6 Synthesis of 1-butyl-3-methylimidazolium bis(trifluoromethanesulfonyl)imide, $[C_4C_1Im][NTf_2]$

$[C_4C_1Im][NTf_2]$ was synthesised based upon the literature.[2] Li$[NTf_2]$ (49 g, 172 mmol) was dissolved into 50 cm^3 of deionised water, and $[C_4C_1Im]Cl$ (29 g, 166 mmol) dissolved into 50 cm^3 of deionised water. Both solutions were mixed, and stirred for 24 hours at room temperature. Dichloromethane (60 cm^3) was added to the mixture, and the mixture was then transferred to a separating funnel. The lower organic phased was collected and washed with water. This was repeated until no traces of halide anion could be observed (tested with silver nitrate solution). The solvent was removed by vacuum giving a colourless liquid. The yield was 50 g (72 %).

^{1}H-NMR (400 MHz, DMSO$-D_6$) δ (ppm): 9.47 (1H, s, N$-$C**H**$-$N), 7.78 (2H, dd, N$-$C**H**$-$C**H**$-$N), 4.19 (2H, t, N$-$C**H**$_2-CH_3$), 3.87 (3H, s,C**H**$_3-$N), 1.76 (2H,

m,$CH_2-\mathbf{CH_2}-CH_2-CH_3$), 1.23 (2H, m, $CH_2-\mathbf{CH_2}-CH_3$), 0.89 (3H, t, $CH_2-\mathbf{CH_3}$). ^{13}C-NMR (100 MHz, DMSO$-D_6$) δ (ppm): 137.2 (s, N$-$**C**H$-$N), 123.6 (s, N$-$**C**H$-$CH$-$N), 122.7 (s,N$-$CH$-$**C**H$-$N), 118.3 (s, $-\mathbf{C}F_3$), 48.5 (s, N$-\mathbf{C_2}$), 35.7 (s,N$-\mathbf{C}H_3$), 31.3 (s, N$-CH_2-\mathbf{C}H_2$), 19.1 (s,$-CH_2-\mathbf{C}H_2-CH_3$), 13.2 (s, $-CH_2-\mathbf{C}H_3$).

2 Characterisation

2.1 Density and viscosity

The densities of the three ionic liquids: $[C_4C_1Im][NTf_2]$, $[C_4^4CNPy][NTf_2]$ and $[C_4^3CNPy][NTf_2]$, and the mixture $[C_4^4CNPy]_{0.5}[C_4C_1Im]_{0.5}[NTf_2]$ were measured between 293–333 K at atmospheric pressure. The data were fitted to linear functions of temperature, the average relative deviation of the fits being always better than 0.01%:

$$\rho[[C_4C_1Im][NTf_2]]/g\ cm^{-3} = 1.7226 - 0.00096 \times (T/\mathrm{K}) \quad (1)$$

$$\rho[[C_4^4CNPy][NTf_2]]/g\ cm^{-3} = 1.7481 - 0.00090 \times (T/\mathrm{K}) \quad (2)$$

$$\rho[[C_4^3CNPy][NTf_2]]/g\ cm^{-3} = 1.7434 - 0.00088 \times (T/\mathrm{K}) \quad (3)$$

$$\rho[[C_4^4CNPy]_{0.5}[C_4C_1Im]_{0.5}[NTf_2]]/g\ cm^{-3} = 1.7360 - 0.00094 \times (T/\mathrm{K}) \quad (4)$$

Table S3: Experimental values for densities (ρ/ g cm^{-3}) for the ionic liquids with constant anion but differing cations to study the impact of the cyanopyridnium cation ($[NTf_2]^-$), for temperatures between 293–333 K and at 0.1 MPa pressure.

Temperature (K)	$[C_4^4CNPy][NTf_2]$	$[C_4^3CNPy][NTf_2]$	$[C_4C_1Im][NTf_2]$
293	1.4843	1.4863	1.4413
303	1.4753	1.4769	1.4364
313	1.4662	1.4676	1.4316
323	1.4572	1.4597	1.4269
333	1.4483	1.4510	1.4221
Temperature (K)	$[C_4^4CNPy]_{0.25}{}^{+*}$	$[C_4^4CNPy]_{0.5}{}^{+*}$	$[C_4^4CNPy]_{0.75}{}^{+*}$
293	1.4506	1.4613	1.4721
303	1.4411	1.4517	1.4624
313	1.4316	1.4423	1.4530
323	1.4222	1.4330	1.4438
333	1.4129	1.4237	1.4346

$*$ These samples are mixtures of respectively $[C_4^4CNPy][NTf_2]$ with $[C_4C_1Im][NTf_2]$, thus mixture of ionic liquid full notation would be $[C_4^4CNPy]_x[C_4C_1Im]_{(1-x)}[NTf_2]$

Table S4: Experimental values for viscosity (η/mPa$\cdot$s) for the ionic liquids with the same anion ($[NTf_2]^-$) but differing cations to study the impact of the cyanopyridnium cation, for temperatures between 293–333 K and at 0.1 MPa pressure. $[C_4C_1Im][NTf_2]$ is included as a reference.

Temperature (K)	$[C_4C_1Im][NTf_2]$	$[C_4^4CNPy][NTf_2]$	$[C_4^3CNPy][NTf_2]$
293	64.07	967.53	1685.40
303	41.57	429.22	716.61
313	28.54	216.73	348.64
313	28.62	201.23	348.64
323	20.65	122.27	190.04
333	15.49	74.35	111.65
Temperature (K)	$[C_4^4CNPy]_{0.25}{}^{+*}$	$[C_4^4CNPy]_{0.5}{}^{+*}$	$[C_4^4CNPy]_{0.75}{}^{+*}$
293	108.60	198.60	383.40
303	65.31	110.80	195.10
313	42.88	67.34	110.40
313	43.00	67.91	111.20
323	29.80	44.40	68.15
333	21.72	30.82	44.74

$*$ These samples are mixtures of respectively $[C_4^4CNPy][NTf_2]$ with $[C_4C_1Im][NTf_2]$, thus mixture of ionic liquid full notation would be $[C_4^4CNPy]_x[C_4C_1Im]_{(1-x)}[NTf_2]$

Table S5: Vogel-Fulcher-Tammann equation parameters and associated uncertainty at a 95% level of confidence obtained from the fitting of viscosity (η/mPa·s) as a function of temperature (T/K) between 293 and 343 K at 0.1 MPa, together with the average relative deviation of the experimental data points to the fitting.

	η_∞	B	T_0	ARD %
$[C_4^4CNPy][NTf_2]$	0.09	954.63	190.20	2.90
$[C_4^3CNPy][NTf_2]$	0.67	600.38	216.98	1.91
$[C_4C_1Im][NTf_2]$	0.17	747.00	166.56	0.56
$[C_4^4CNPy]_{0.25}{}^{+*}$	0.32	613.83	187.70	0.70
$[C_4^4CNPy]_{0.50}{}^{+*}$	0.20	749.43	184.60	0.74
$[C_4^4CNPy]_{0.75}{}^{+*}$	0.15	834.19	186.69	0.88

∗ These samples are mixtures of respectively $[C_4^4CNPy][NTf_2]$ with $[C_4C_1Im][NTf_2]$, thus mixture of ionic liquid full notation would be $[C_4^4CNPy]_x[C_4C_1Im]_{(1-x)}[NTf_2]$.

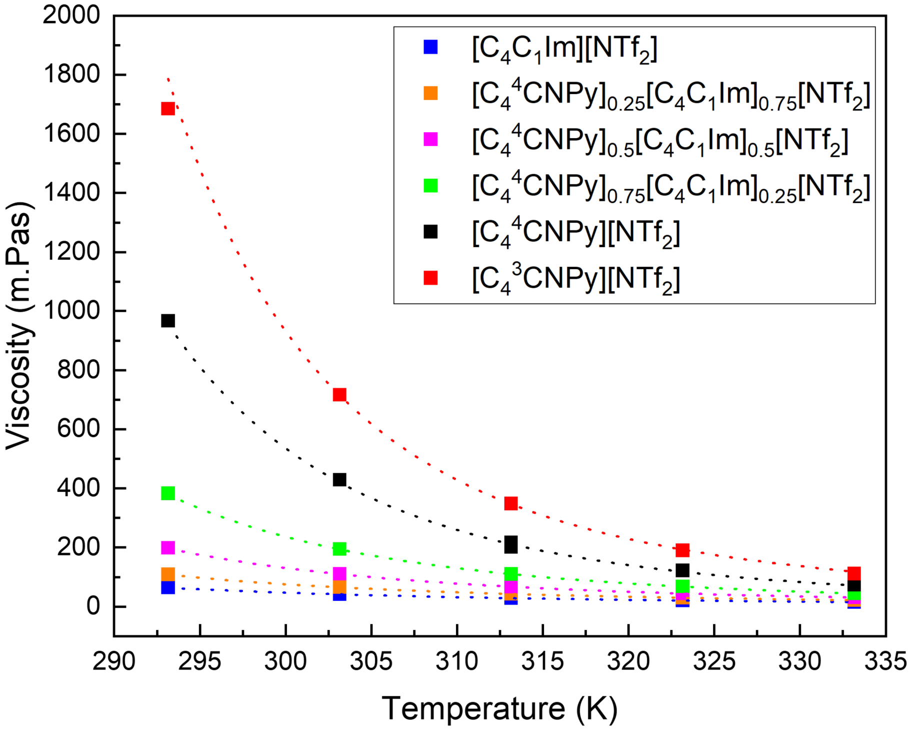

Figure S1: Experimental viscosities (η/mPa·s) of the ionic liquids studied against temperature (K), the lines on the experimental data represent the fitting of the VFT parameters as seen in the manuscript.

Table S6: Effect of temperature on the value of G for the mixture of $[C_4C_1Im][NTf_2]$ + $[C_4^4CNPy][NTf_2]$ from 293.15 K to 303.15 K. G is related to the viscosities and mole fraction of the pure ionic liquids and the mixture of the ionic liquids from equation 5

Temperature (K)	G
293.15	-0.44
303.15	-0.37
313.15	-0.29
323.15	-0.24
333.15	-0.19

The ionic liquid-ionic liquid mixtures are fitted with the Grunberg and Nissan equation:

$$log(\mu_m) = x_1 log(\mu_1) + x_2 log(\mu_2) + x_1 x_2 G \tag{5}$$

2.2 Thermal stability

Thermal analysis of materials was performed using a TA Instruments Q5000 thermogravimetric analyser. Weight uncertainty is 0.1 μ g. The method used for both the [C_4^3CNPy][NTf_2] and [C_4^3CNPy][NTf_2] is shown in table S7, and their respective profiles in figure S2 and figure S3.

Table S7: TGA method used utilised for [C_4^3CNPy][NTf_2] and [C_4^3CNPy][NTf_2]

Steps	Start temp (°C)	End temp(°C)	Ramp (°C/min)	Hold time (min)
1	30	600	10	
2	600	600	0	5

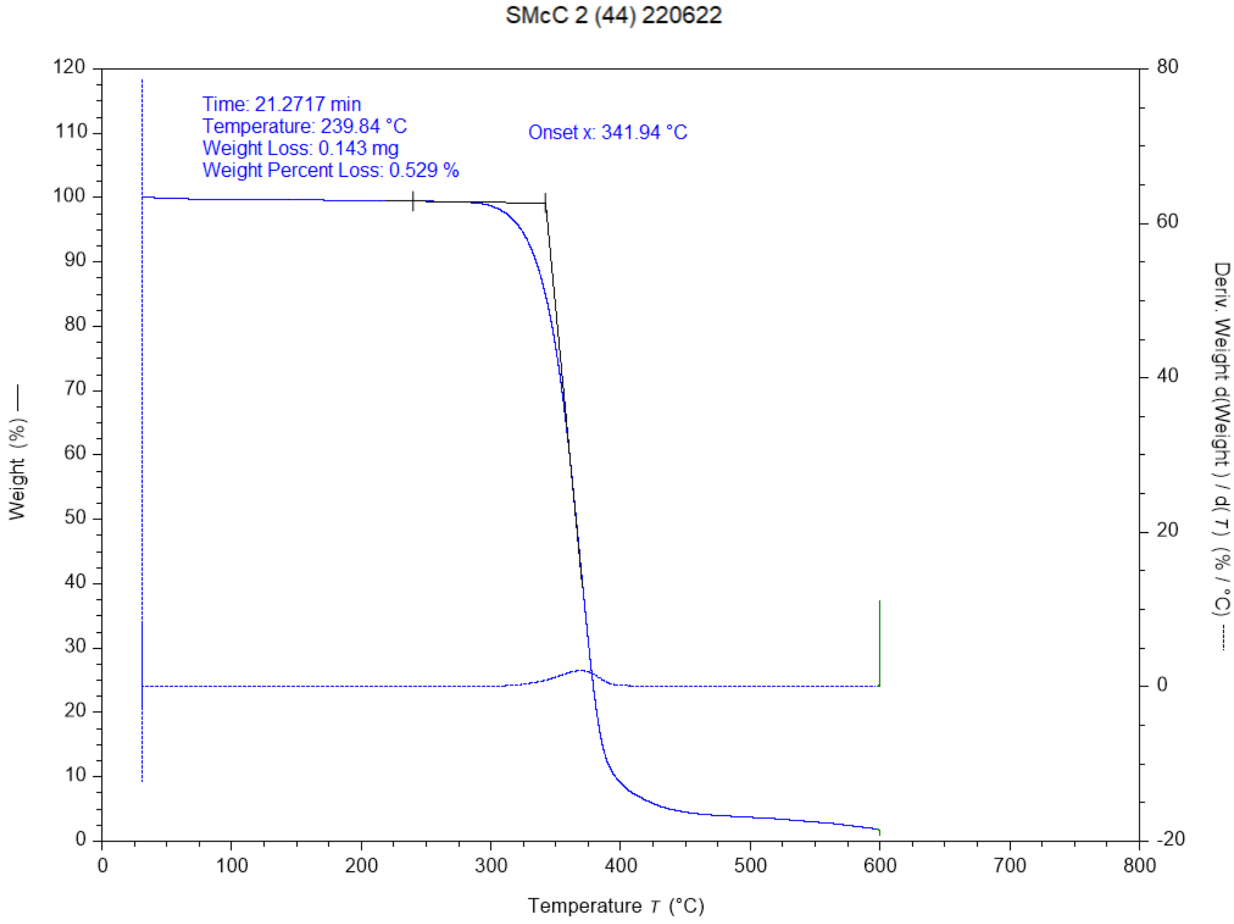

Figure S2: TGA of [C_4^4CNPy][NTf_2]

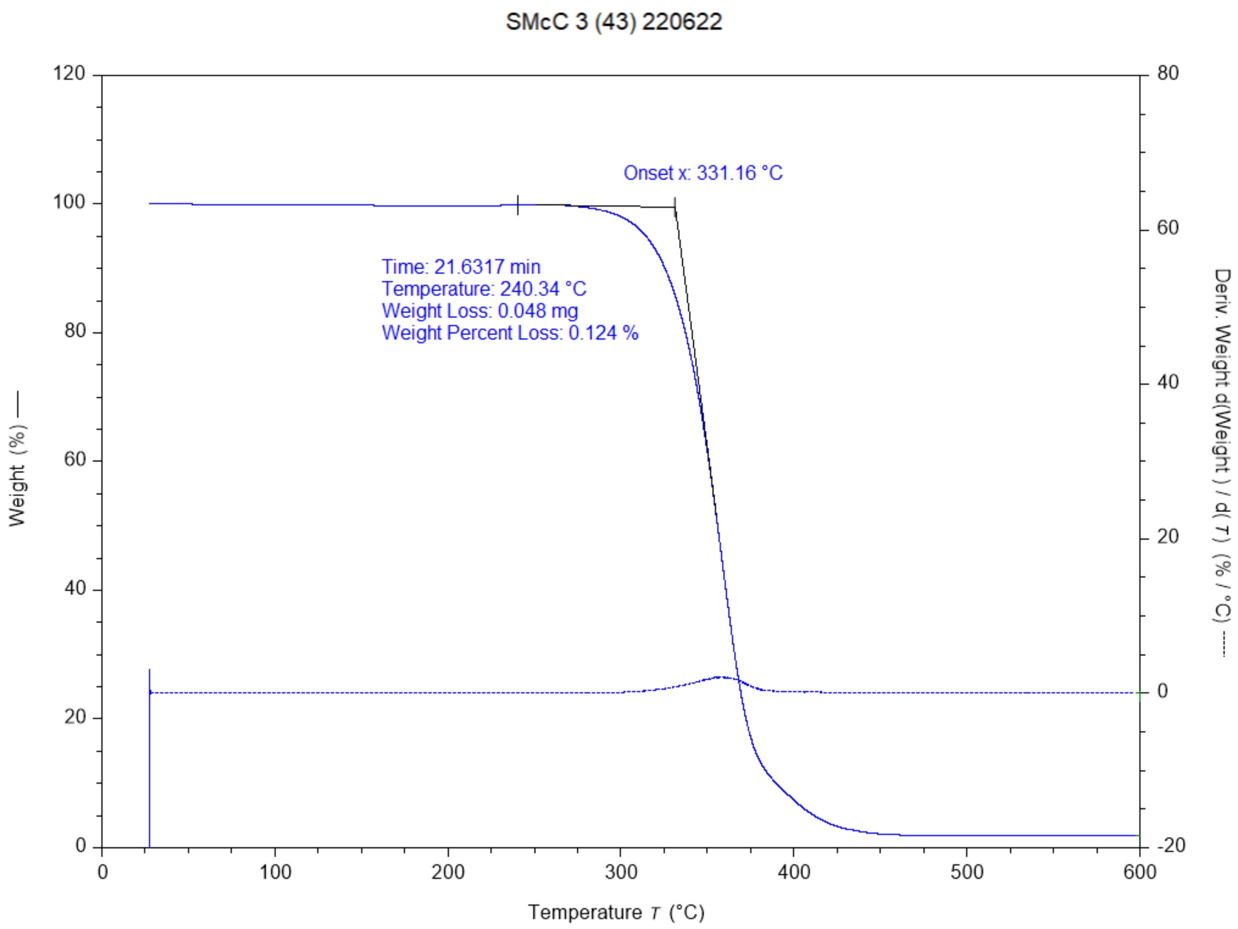

Figure S3: TGA of $[C_4^3CNPy][NTf_2]$

2.3 Phase behaviour

Differential scanning calorimetry, using a TA Instruments Q2000 DSC was used to investigate the phase behaviour of the ionic liquids. The method used for both the [C_4^4CNPy][NTf_2] and [C_4^3CNPy][NTf_2] is shown in table S8. The uncertanity associated with the TA instrument Q2000 is ±0.1°C, and calorimetric reproducibility (with indium metal) is ±0.05 %. The [C_4^4CNPy][NTf_2] DSC profile in figure S4 and the [C_4^3CNPy][NTf_2] DSC profile in figure S5. The glass transition temperature values, Tg, are calculated by manual peak analysis, so it is difficult to determine the associated error.

Table S8: DSC method used for both ionic liquids

Steps	Start temp (°C)	End temp(°C)	Ramp (°C/min)	Hold time (min)
1	20	-70	5	
2	-70	-70	0	5
3	-70	50	5	
4	Repeat steps 1 (50°C instead of 20°C going forward) to 3 twice			

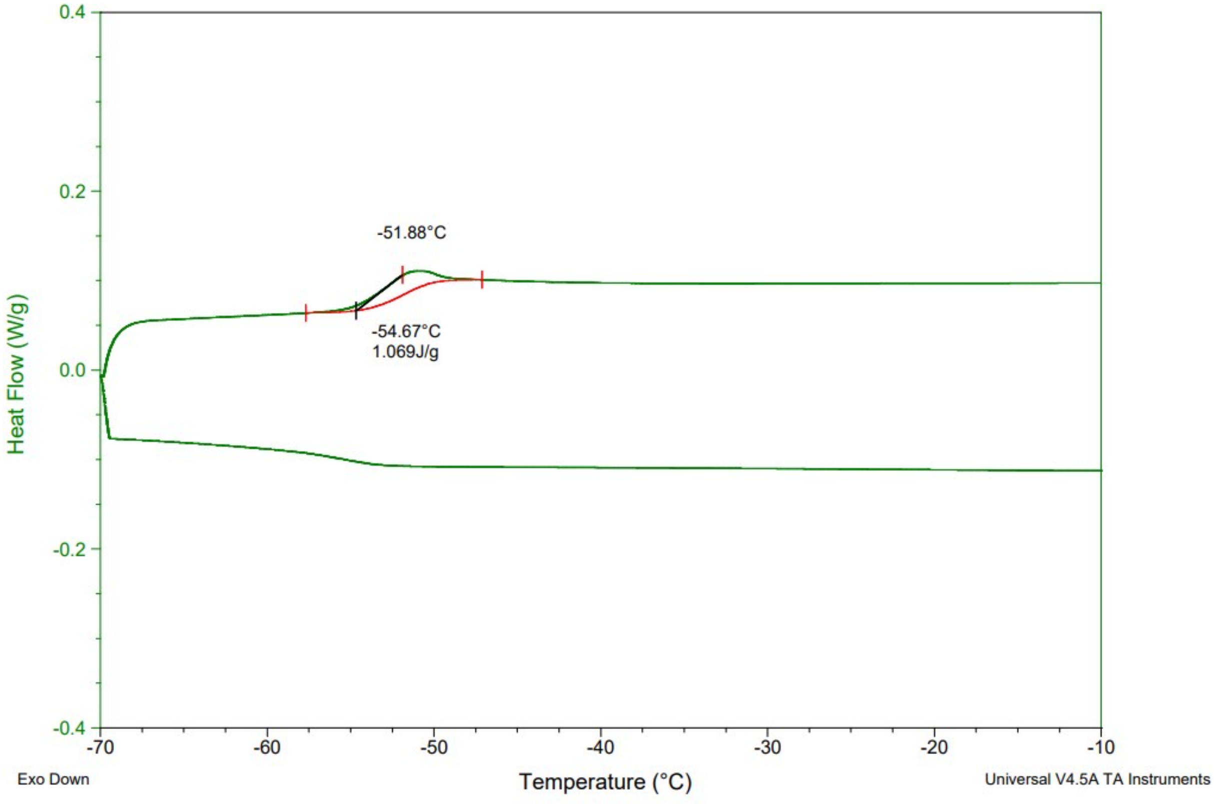

Figure S4: DSC of [C_4^4CNPy][NTf_2] (exothermic down)

2.4 Mass spectrometry

The high-resolution mass spectra were recorded in positive and negative mode on a hybrid quadrupole time of flight (TOF) mass spectrometer (Waters Xevo G2-XS QTof) with an Electrospray Ionisation (ESi) source. The gas flow of spray gas is 50 L/Hour, capillary voltage : +/- 3.0 kV. Cone voltage : 50V and source temperature : 150 °C Mass range: 20-1600 m/z for positive and negative modes.

3 2D NMR

^{1}H diffusion measurements (DOSY) were carried out applying a stimulated-echo NMR pulse sequence (using ledbpgp2s) with the delay for gradient recovery (d16): 0.2 ms, duration of the

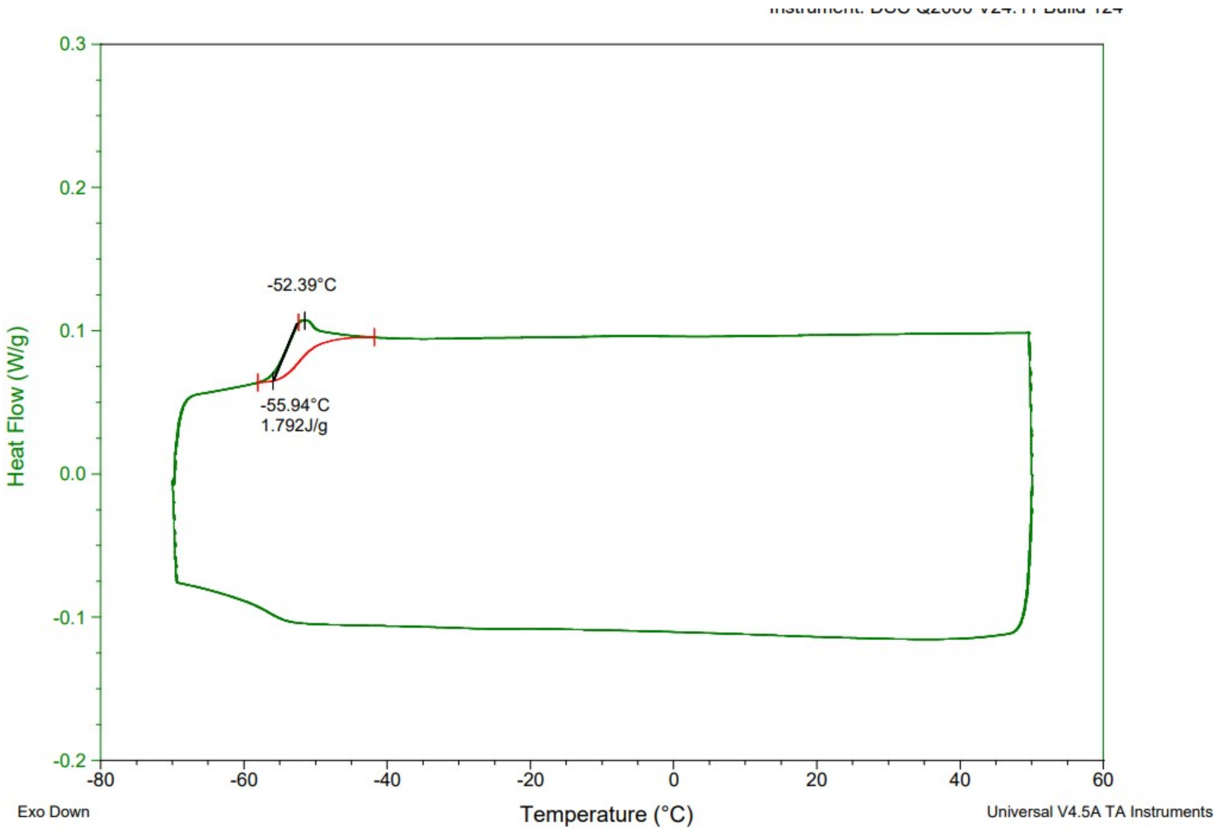

Figure S5: DSC of [C_4^3CNPy][NTf_2] (exothermic down)

gradient purge pulse (p19): 0.6 ms, the total diffusion-encoding pulse duration (p30): 1.8 ms and diffusion delay D (d20): 0.1 s. Each FID was acquired using 32 k data points.

NOESY experiments were carried using noesygpph (phase senstive) with gradient pulses in mixing time at 300 ms and a pulse sequence of 45G/m.

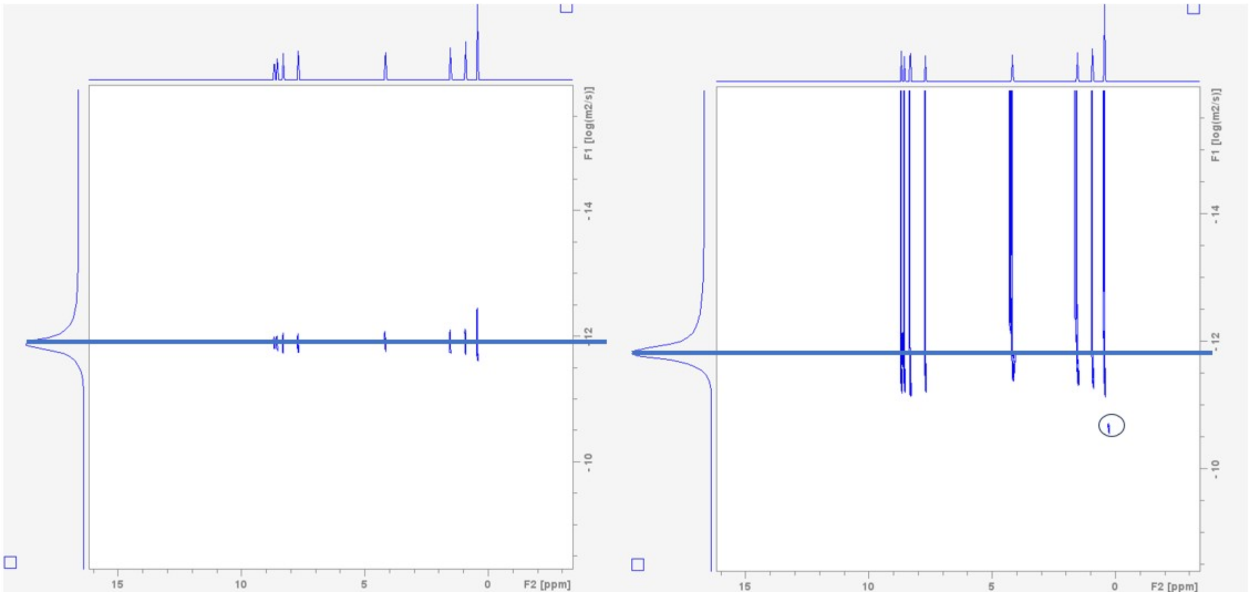

Figure S6: DOSY NMR of $[C_4^3CNPy][NTf_2]$ (left) and $[C_4^3CNPy][NTf_2]$ (right) containing ethane. The ethane peak is highlighted by the circles.

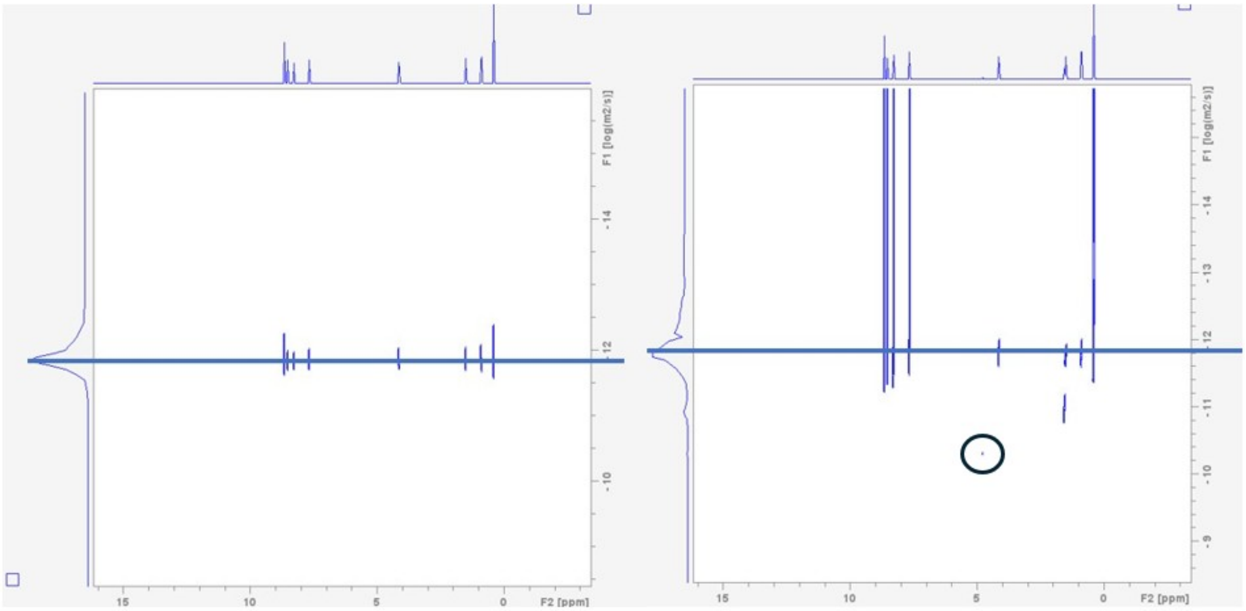

Figure S7: DOSY NMR of $[C_4^3CNPy][NTf_2]$ (left) and $[C_4^3CNPy][NTf_2]$ (right) containing ethylene. The ethylene peak is highlighted by the circles.

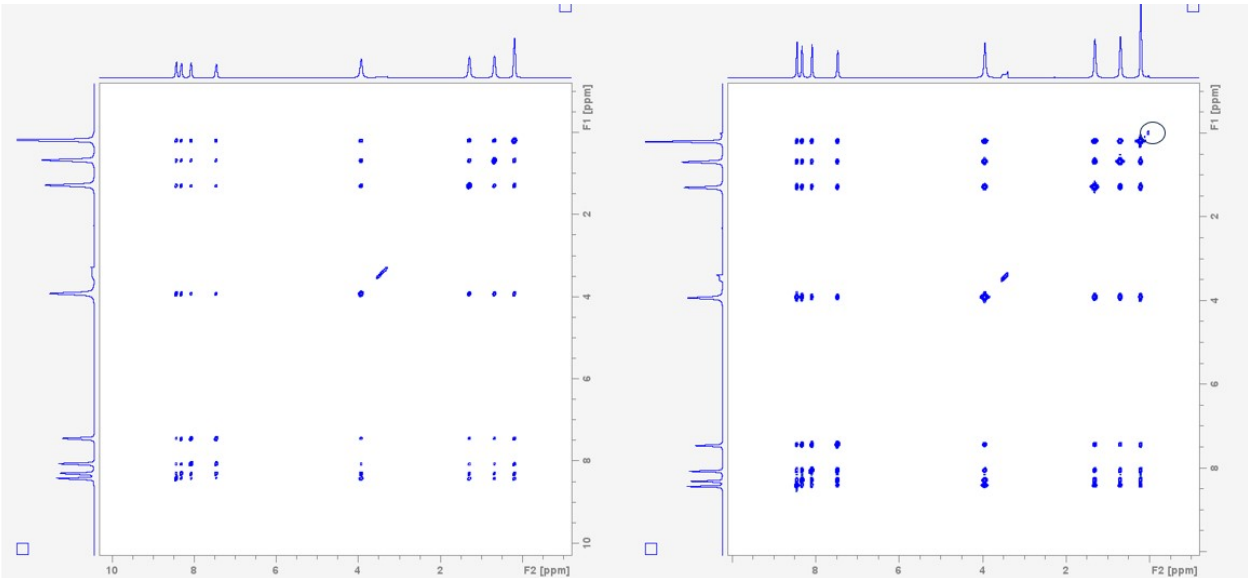

Figure S8: NOESY NMR of $[C_4^3CNPy][NTf_2]$ (left) and $[C_4^3CNPy][NTf_2]$ (right) containing ethane. The ethane peak is highlighted by the circles.

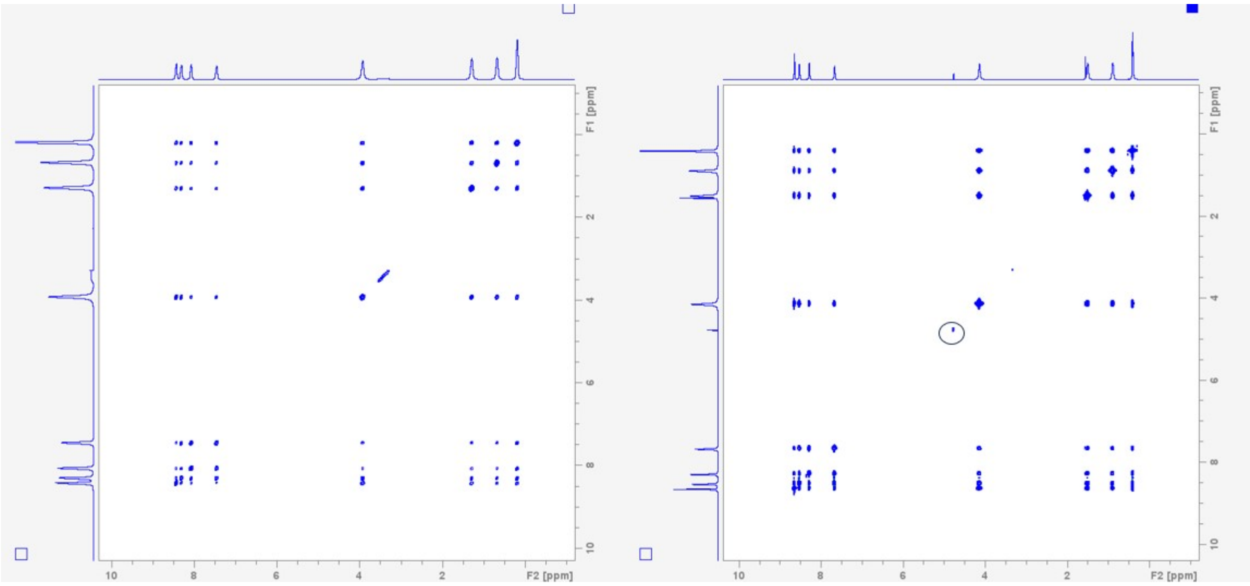

Figure S9: NOESY NMR of $[C_4^3CNPy][NTf_2]$ (left) and $[C_4^3CNPy][NTf_2]$ (right) containing ethylene. The ethylene peak is highlighted by the circles.

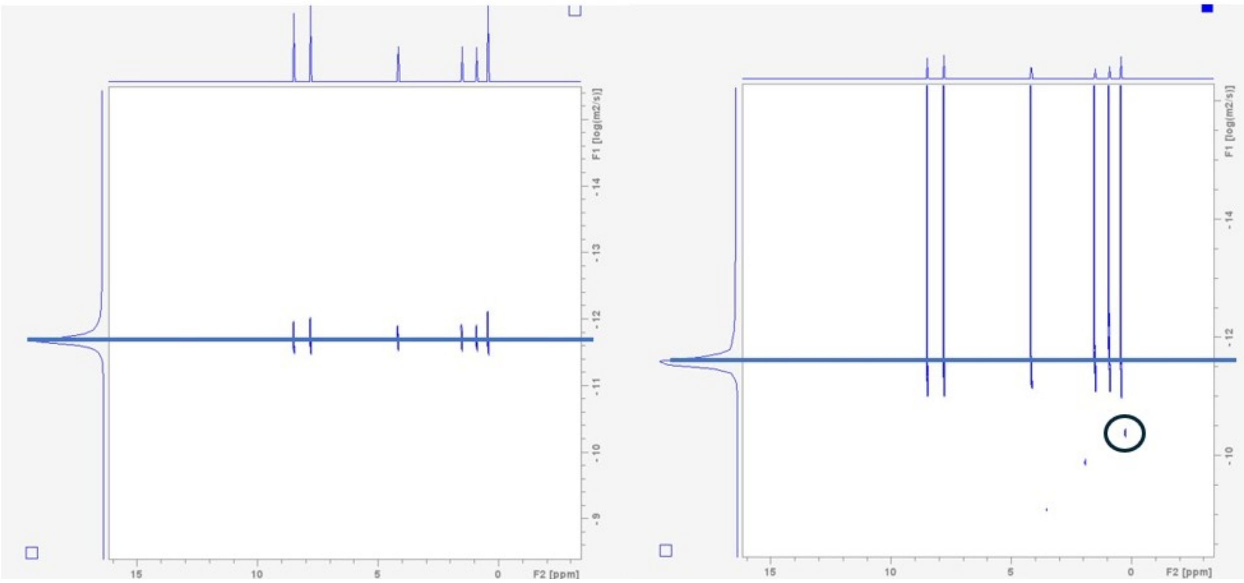

Figure S10: DOSY NMR of [C_4^4CNPy][NTf_2] (left) and [C_4^4CNPy][NTf_2] (right) containing ethane. The ethane peak is highlighted by the circles.

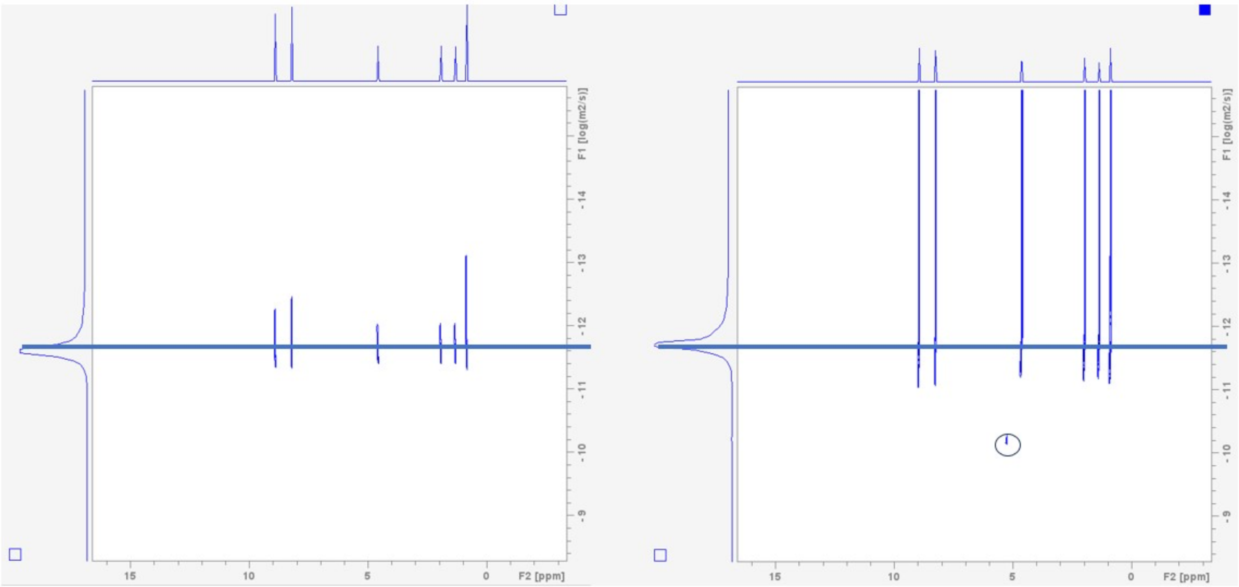

Figure S11: DOSY NMR of [C_4^4CNPy][NTf_2] (left) and [C_4^4CNPy][NTf_2] (right) containing ethylene. The ethylene peak is highlighted by the circles.

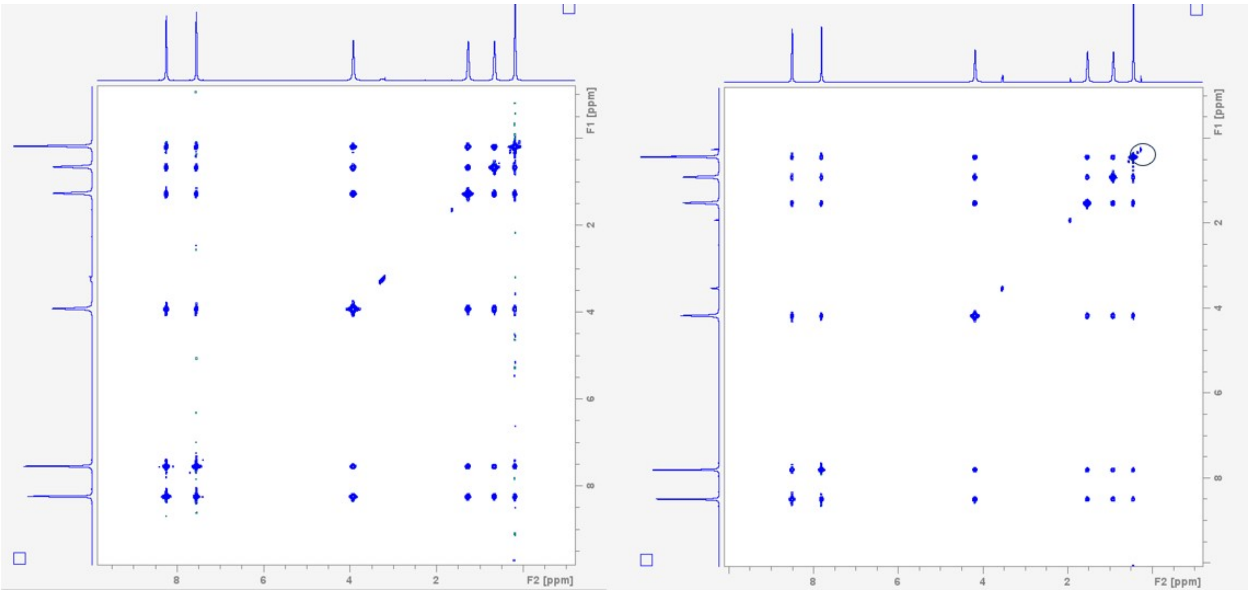

Figure S12: NOESY NMR of $[C^4_4CNPy][NTf_2]$ (left) and $[C^4_4CNPy][NTf_2]$ (right) containing ethane. The ethane peak is highlighted by the circles.

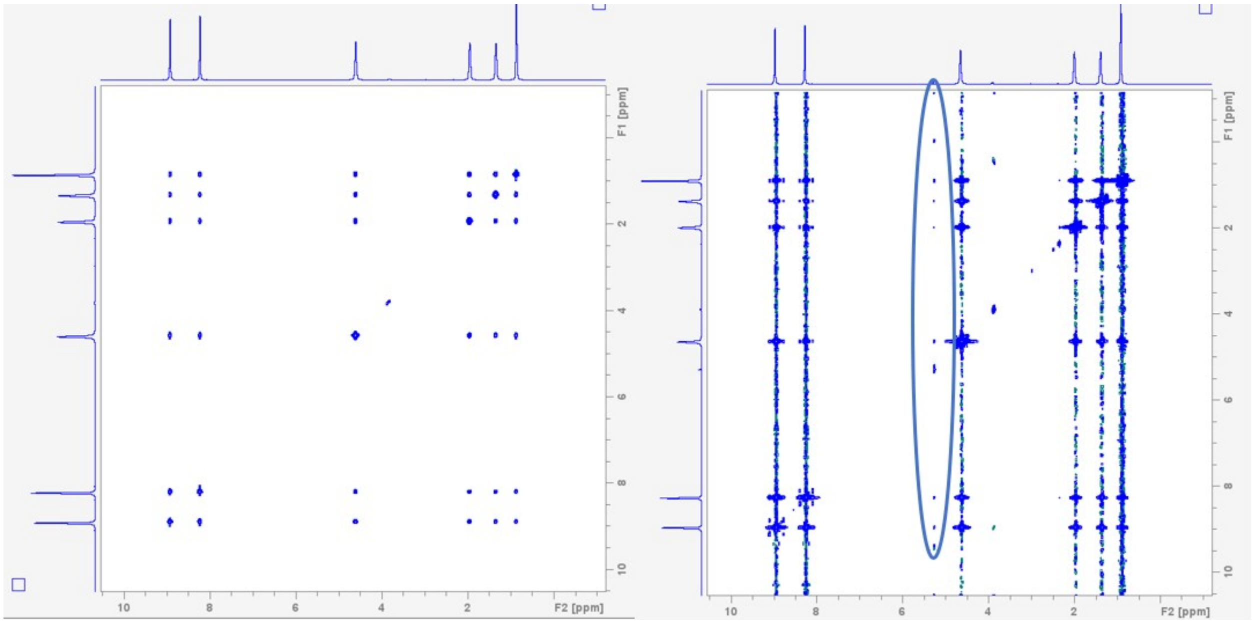

Figure S13: NOESY NMR of $[C^4_4CNPy][NTf_2]$ (left) and $[C^4_4CNPy][NTf_2]$ (right) containing ethylene. The ethylene peak is highlighted by the circles.

4 Gas solubility

4.1 Gas solubility system (GSS)

The GSS was commissioned with C_2H_4 in $[C_4C_1Im][NTf_2]$.[3–6] The experimental gas solubility including the mole fraction and Henry's Law constant data for C_2H_4 in $[C_4C_1Im][NTf_2]$ is shown in table S9, alongside the temperature and equilibrated pressure of the system.

Table S9: Raw experimental values for the solubility of ethylene in $[C_4C_1Im][NTf_2]$, expressed as Henry's Law constants(K_H), and as gas mole fraction (x_{gas}). Temperature is denoted as T and the experimental equilibrium pressure is denoted as P.

Gas	Material	T (K)	P (mbar)	K_H (bar)	x_{gas}
C_2H_4	$[C_4C_1Im][NTf_2]$	303.15	880	73	0.0121
		313.15	935	106	0.0088
		323.15	980	150	0.0065
		333.15	1022	237	0.0043

The experimental solubility data has been corrected for 0.1 MPa, and compared to the published literature. Anthony *et al.* also studied this solubility but again at 25°C, and found a mole fraction of 0.013 (a 8% deviation from this work). A higher mole fraction at lower temperature would be expected, with increasing temperature decreasing the gas solubility in the sorbent.[3] Moura and Xing both found a mole fraction of 0.013 (8% deviation) at 30 °C. Both Moura and Xing used a liquid bath to maintain the constant temperature compared to the heating tape used in the GSS. This would allow for greater temperature control.[4,7] Palgunadi determined a similar value of mole fraction as this work, albeit at 40 °C,[6] suggesting poorer temperature control although an isothermal oven was used. The oven would be more similar with GSS in terms of heat transfer to the gas, through convection in the air compared to a liquid media.[6] Zhang presented a smaller mole fraction of ethylene (a 9% deviation) used the GC with a ionic liquid packed column, compared to the gravimetric and PVT.[5] This method is more intrusive compared to the gravimetric or PVT methods.

Table S10: The mole fraction of CO_2 in $[C_4C_1Im][NTf_2]$ using the GSSB corrected to 1 bar and the corresponding solubility from other reported work. The deviation between this work and reported literature are shown alongside with the temperature the solubility was determined and the solubility method. The deviation is the difference between this work, and the reported literature at the same equilibrated pressure.

Source	$x_{C_2H_4}$ at 1 bar	deviation (%)	T (°C)	Method
This work	0.012		30	PVT
Anthony[3]	0.013	7.69	25	Gravimetric
Moura[7]	0.013	7.69	30	PVT
Xing[4]	0.013	7.69	30	PVT
Palgunadi[6]	0.012	0.00	40	PVT
Zhang[5]	0.011	-9.09	30	GC set-up

The experimental mole fraction of ethylene from this work, and literature data are plotted in figure S14 up to 6 bar, and a generalised linear trend can be seen with some anomaly data from Anthony.[3]

The uncertainty of the GSS has been determined as 7.26% of the output mole fraction of gas in the sorbent. The relevant uncertainties (excluding minimal uncertainties such as determining the mass and density of sorbent) occur from the pressure transmitters (0.1%), temperature measurement in the reactor (0.5%), and the two hot plates used (6.67% total).

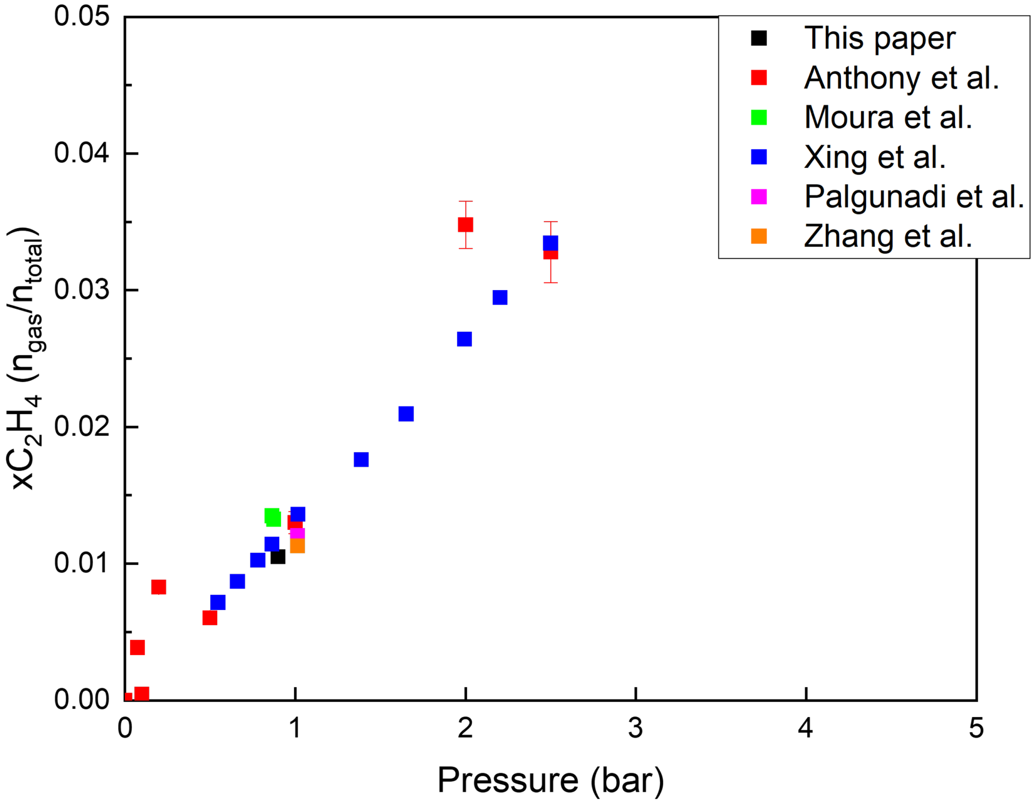

Figure S14: Mole fraction of C_2H_4 in $[C_4C_1Im][NTf_2]$ as a function of equilibrated pressure at 30 °C for this work. From literature, ■ Anthony (25 °C), ■ Moura (30 °C), ■ Xing (30 °C), ■ Palgunadi (40 °C) and ■ Zhang (30 °C) are included for comparison.[3–7]

4.2 Pure gas solubility data

Table S11: Experimental data for the solubility of ethylene in $[C_4^4CNPy][NTf_2]$, expressed as Henry's Law constants (K_H), and as gas mole fraction (x_{gas}) at the equilibrated pressure, and as a gas mole fraction, x_2 corrected for a partial pressure of solute of 0.1 MPa. Temperature is denoted as T and the experimental equilibrium pressure is denoted as P.

T (K)	P (mbar)	K_H (bar)	$x_{ethylene}$	%dev	x_2
303.15	1616	92	0.017	7.10	0.0108
303.15	1110	111	0.010	-11.13	0.0090
303.15	903	118	0.008	-18.01	0.0085
313.15	1674	102	0.016	10.69	0.0098
313.15	1154	130	0.009	-12.96	0.0077
313.15	934	132	0.007	-15.32	0.0076
323.15	1739	119	0.014	8.03	0.0084
323.15	1199	155	0.008	-19.90	0.0064
323.15	970	157	0.006	-21.47	0.0064
333.15	1790	128	0.014	11.23	0.0078
333.15	1240	181	0.007	-25.76	0.0055
333.15	999	173	0.006	-20.69	0.0058

Table S12: Experimental data for the solubility of ethane in $[C_4^4CNPy][NTf_2]$, expressed as Henry's Law constants (K_H), and as gas mole fraction (x_{gas}) at the equilibrated pressure, and as a gas mole fraction, x_2 corrected for a partial pressure of solute of 0.1 MPa. Temperature is denoted as T and the experimental equilibrium pressure is denoted as P.

T (K)	P (mbar)	K_H (bar)	x_{ethane}	%dev	x_2
303.15	1634	127	0.013	6.72	0.0079
303.15	1179	160	0.007	-17.37	0.0063
303.15	920	137	0.007	-0.57	0.0073
313.15	1682	134	0.012	8.42	0.0075
313.15	1209	163	0.007	-11.21	0.0061
313.15	980	146	0.006	0.25	0.0068
323.15	1740	152	0.011	5.86	0.0066
323.15	1249	185	0.007	-14.77	0.0054
323.15	980	168	0.006	-4.49	0.0059
333.15	1792	166	0.011	8.19	0.0060
333.15	1287	206	0.006	-13.91	0.0049
333.15	1012	194	0.005	-7.33	0.0051

Table S13: Experimental data for the solubility of ethylene in $[C_4^3CNPy][NTf_2]$, expressed as Henry's Law constants (K_H), and as gas mole fraction (x_{gas}) at the equilibrated pressure, and as a gas mole fraction, x_2 corrected for a partial pressure of solute of 0.1 MPa. Temperature is denoted as T and the experimental equilibrium pressure is denoted as P.

T (K)	P (mbar)	K_H (bar)	$x_{ethylene}$	%dev	x_2
303.15	1160	133	0.009	-0.90	0.0075
303.15	926	137	0.007	-3.58	0.0073
303.15	1520	129	0.012	2.54	0.0078
313.15	1193	138	0.009	1.88	0.0072
313.15	954	146	0.006	-3.90	0.0068
313.15	1567	137	0.011	2.71	0.0073
323.15	1234	156	0.008	-2.17	0.0064
323.15	992	177	0.006	-16.01	0.0056
323.15	1612	145	0.011	5.18	0.0069
333.15	1271	170	0.007	-0.71	0.0059
333.15	1013	172	0.006	-1.96	0.0058
333.15	1667	165	0.010	2.46	0.0061

Table S14: Experimental data for the solubility of ethane in $[C_4^3CNPy][NTf_2]$, expressed as Henry's Law constants (K_H), and as gas mole fraction (x_{gas}) at the equilibrated pressure, and as a gas mole fraction, x_2 corrected for a partial pressure of solute of 0.1 MPa. Temperature is denoted as T and the experimental equilibrium pressure is denoted as P.

T (K)	P (mbar)	K_H (bar)	x_{ethane}	%dev	x_2
303.15	970	174	0.006	-4.54	0.0057
303.15	1477	153	0.010	7.87	0.0065
313.15	1216	185	0.007	-5.46	0.0054
313.15	1004	200	0.005	-14.17	0.0050
313.15	1568	162	0.009	7.60	0.0062
323.15	1254	202	0.006	-2.40	0.0049
323.15	1040	243	0.004	-22.86	0.0041
323.15	1568	176	0.009	10.75	0.0057
333.15	1299	249	0.005	-5.63	0.0040
333.15	1070	262	0.004	-11.08	0.0038
333.15	1629	220	0.007	6.80	0.0045

Table S15: Experimental data for the solubility of ethylene in $[C_4^4CNPy]_{0.5}[C_4C_1Im]_{0.5}[NTf_2]$, expressed as Henry's Law constants (K_H), and as gas mole fraction (x_{gas}) at the equilibrated pressure, and as a gas mole fraction, x_2 corrected for a partial pressure of solute of 0.1 MPa. Temperature is denoted as T and the experimental equilibrium pressure is denoted as P.

T (K)	P (mbar)	K_H (bar)	$x_{ethylene}$	%dev	x_2
303.15	1101	84	0.013	4.04	0.0119
303.15	903	94	0.010	-7.70	0.0106
303.15	1210	87	0.014	0.28	0.0115
313.15	1145	94	0.012	5.00	0.0106
313.15	930	99	0.009	0.30	0.0101
313.15	1258	100	0.013	-0.32	0.0100
323.15	1191	109	0.011	2.60	0.0092
323.15	969	116	0.008	-3.82	0.0086
323.15	1304	114	0.011	-1.62	0.0088
333.15	1231	120	0.010	4.08	0.0083
333.15	1001	129	0.008	-2.58	0.0078
333.15	1347	126	0.011	-0.46	0.0079

Table S16: Experimental data for the solubility of ethane in $[C_4^4CNPy]_{0.5}[C_4C_1Im]_{0.5}[NTf_2]$, expressed as Henry's Law constants (K_H), and as gas mole fraction (x_{gas}) at the equilibrated pressure, and as a gas mole fraction, x_2 corrected for a partial pressure of solute of 0.1 MPa. Temperature is denoted as T and the experimental equilibrium pressure is denoted as P.

T (K)	P (mbar)	K_H (bar)	x_{ethane}	%dev	x_2
303.15	1060	122	0.009	3.59	0.0082
303.15	924	121	0.008	4.79	0.0083
303.15	1165	134	0.009	-5.32	0.0075
313.15	1099	138	0.008	5.14	0.0072
313.15	957	136	0.007	6.65	0.0073
313.15	1249	152	0.008	-4.38	0.0066
323.15	1139	160	0.007	4.21	0.0063
323.15	992	157	0.006	6.06	0.0064
323.15	1249	180	0.007	-7.87	0.0056
333.15	1180	188	0.006	1.21	0.0053
333.15	1025	177	0.006	7.20	0.0057
333.15	1288	200	0.006	-5.25	0.0050

Table S17: Parameters A_i used to fit the experimental results as a function of the Henry's Law constants, K_H as well as, the average relative deviation of the experimental points to the fit.

		A_0	A_1	A_2	ARD %
$[C_4^4CNPy][NTf_2]$	Ethylene	3.59	2.00E+03	-5.12E05	15.2
	Ethane	21.98	-9.81E+03	1.40E+06	8.3
$[C_4^3CNPy][NTf_2]$	Ethylene	19.03	-8.10E+03	1.16E+06	3.7
	Ethane	44.78	-2.40E+04	3.62E+06	8.9
$[C_4^4CNPy]_{0.5}[C_4C_1Im]_{0.5}[NTf_2]$	Ethylene	8.64	-1.32E+03	1.71E+04	2.7
	Ethane	11.65	-2.83E+03	2.33E+05	5.1

4.3 Mixed gas solubility data

Table S18: Raw experimental values for the solubility of ethylene and ethane (as a 50:50% molar mixture) in [C_4^4CNPy][NTf_2], expressed as Henry's Law constants (K_H), and as gas mole fraction (x_{gas}). Temperature is denoted as T and the experimental equilibrium pressure is denoted as P.

T (K)	P (mbar)	K_H (bar)	$x_{ethylene}$
303.15	940.49	764.11	0.0034
303.15	1366.00	776.18	0.0048
303.15	1221.35	772.42	0.0043
T(K)	P (mbar)	K_H (bar)	x_{ethane}
303.15	940.49	754.01	0.0034
303.15	1366.00	765.92	0.0049
303.15	1221.35	762.21	0.0044

Table S19: Thermodynamic functions of solvation of the gases studied in $[C_4^4CNPy][NTf_2]$ at several temperatures between 303 and 333 K.

Temperature (K)	$\Delta_{solv}G^{\infty}$/kJ mol^{-1}	$\Delta_{solv}H^{\infty}$ /kJ mol^{-1}	$\Delta_{solv}S^{\infty}$ /J mol^{-1} K^{-1}
C_2H_4			
303.2	11.6	-11.5	-76.2
313.2	12.3	-10.6	-73.3
323.2	13.1	-9.8	-70.7
333.2	13.8	-9.0	-68.3
C_2H_6			
303.2	12.4	-4.5	-55.7
313.2	13.0	-7.0	-63.7
323.2	13.7	-9.3	-71.0
333.2	14.4	-11.4	-77.6

Table S20: Thermodynamic functions of solvation of the gases studied in $[C_4^3CNPy][NTf_2]$ at several temperatures between 303 and 333 K.

Temperature (K)	$\Delta_{solv}G^{\infty}$/kJ mol^{-1}	$\Delta_{solv}H^{\infty}$ /kJ mol^{-1}	$\Delta_{solv}S^{\infty}$ /J mol^{-1} K^{-1}
C_2H_4			
303.2	12.3	-4.0	-53.7
313.2	12.9	-6.0	-60.2
323.2	13.5	-7.9	-66.2
333.2	14.2	-9.7	-71.6
C_2H_6			
303.2	12.9	-0.8	-45.2
313.2	13.4	-7.1	-65.8
323.2	14.2	-13.1	-84.4
333.2	15.1	-18.7	-101.5

Table S21: Thermodynamic functions of solvation of the gases studied in $[C_4^4CNPy]_{0.5}[C_4C_1Im]_{0.5}[NTf_2]$ at several temperatures between 303 and 333 K.

Temperature (K)	$\Delta_{solv}G^{\infty}$/kJ mol^{-1}	$\Delta_{solv}H^{\infty}$ /kJ mol^{-1}	$\Delta_{solv}S^{\infty}$ /J mol^{-1} K^{-1}
C_2H_4			
303.2	11.3	-10.0	-70.3
313.2	12.0	-10.1	-70.4
323.2	12.7	-10.1	-70.5
333.2	13.4	-10.1	-70.5
C_2H_6			
303.2	12.2	-10.8	-75.8
313.2	13.0	-11.2	-77.1
323.2	13.7	-11.6	-78.3
333.2	14.5	-11.9	-79.4

4.4 Calorimetry

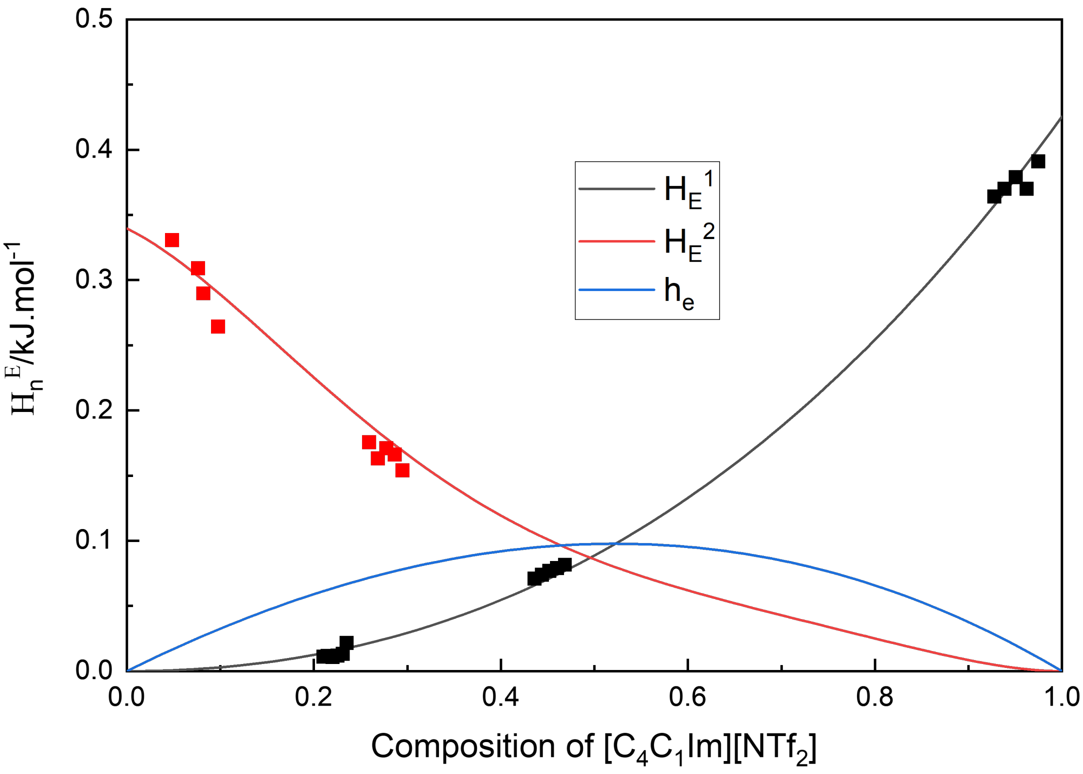

Figure S15: The partial molar excess enthalpies of $[C_4C_1Im][NTf_2]$ (red line) and $[C_4^4CNPy][NTf_2]$ (black line). The molar enthalpy of mixing, $\Delta_{mix}H$, of the mixture of $[C_4C_1Im][NTf_2]$ and $[C_4^4CNPy][NTf_2]$ shown in the blue line, h_e. The experimental data can be found in table S22. The parameters A_i, in the Redlich-Kister equation, obtained by fitting experimental data of partial molar excess enthalpies are, in J.mol^{-1}: $A_0 = 354 \pm 7$, $A_1 = -29 \pm 6$, $A_2 = 63 \pm 13$.

Table S22: Stoichiometric data and heat effects of the calorimetry experiments with the system $[C_4^4CNPy][NTf_2]$ (1) + $[C_4C_1Im][NTf_2]$ (2) at 303.15 K. The subscripts c and d mean cell (or container) and dispenser (or syringe), respectively. Injections 1-4 $[C_4C_1Im][NTf_2]$ in $[C_4^4CNPy][NTf_2]$, injections 5-9 $[C_4^4CNPy][NTf_2]$ in $[C_4C_1Im][NTf_2]$, injections 10-14 $[C_4^4CNPy][NTf_2]$ in 50:50 mixture of ionic liquids, injections 15-20 $[C_4^4CNPy][NTf_2]$ in 25% $[C_4C_1Im][NTf_2]$: 75% $[C_4^4CNPy][NTf_2]$, and injections 21-25 $[C_4C_1Im][NTf_2]$ in 25% $[C_4C_1Im][NTf_2]$: 75% $[C_4^4CNPy][NTf_2]$. Each injection lasted between 3 and 8 min, depending on the composition and the volume of the injection.

	In the cell				In the syringe				
Inj.	x1	x2	n1c/mol	n2c/mol	V1d/μl	n1d/mmol	V2d/μl	n2d/mmol	Q/J
1	0.95	0.05	2.69E-03	1.37E-04	0	0.00E+00	15	5.14E-02	1.70E-02
2	0.95	0.05	2.69E-03	2.22E-04	0	0.00E+00	10	3.43E-02	1.06E-02
3	0.93	0.07	2.69E-03	2.40E-04	0	0.00E+00	15	5.14E-02	1.49E-02
4	0.92	0.08	2.69E-03	2.91E-04	0	0.00E+00	5	1.71E-02	4.52E-03
5	0.01	0.99	6.70E-05	2.57E-03	10	3.35E-02	0	0.00E+00	1.31E-02
6	0.03	0.97	1.01E-04	2.57E-03	10	3.35E-02	0	0.00E+00	1.24E-02
7	0.04	0.96	1.34E-04	2.57E-03	10	3.35E-02	0	0.00E+00	1.27E-02
8	0.05	0.95	1.68E-04	2.57E-03	10	3.35E-02	0	0.00E+00	1.24E-02
9	0.06	0.94	2.01E-04	2.57E-03	10	3.35E-02	0	0.00E+00	1.22E-02
10	0.52	0.48	1.44E-03	1.27E-03	15	5.03E-02	0	0.00E+00	4.11E-03
11	0.53	0.47	1.49E-03	1.27E-03	15	5.03E-02	0	0.00E+00	3.98E-03
12	0.54	0.46	1.54E-03	1.27E-03	15	5.03E-02	0	0.00E+00	3.87E-03
13	0.55	0.45	1.59E-03	1.27E-03	15	5.03E-02	0	0.00E+00	3.73E-03
14	0.56	0.44	1.64E-03	1.27E-03	15	5.03E-02	0	0.00E+00	3.58E-03
15	0.76	0.24	2.05E-03	6.30E-04	15	5.03E-02	0	0.00E+00	1.09E-03
16	0.76	0.24	2.10E-03	6.30E-04	15	5.03E-02	0	0.00E+00	6.66E-04
17	0.77	0.23	2.16E-03	6.30E-04	20	6.71E-02	0	0.00E+00	7.93E-04
18	0.77	0.23	2.23E-03	6.30E-04	20	6.71E-02	0	0.00E+00	7.18E-04
19	0.78	0.22	2.30E-03	6.30E-04	20	6.71E-02	0	0.00E+00	7.87E-04
20	0.78	0.22	2.37E-03	6.30E-04	20	6.71E-02	0	0.00E+00	7.46E-04
21	0.75	0.25	2.00E-03	6.99E-04	0	0.00E+00	10	3.43E-02	6.02E-03
22	0.74	0.26	2.00E-03	7.33E-04	0	0.00E+00	10	3.43E-02	5.60E-03
23	0.73	0.27	2.00E-03	7.67E-04	0	0.00E+00	10	3.43E-02	5.86E-03
24	0.72	0.28	2.00E-03	8.01E-04	0	0.00E+00	10	3.43E-02	5.70E-03
25	0.71	0.29	2.00E-03	8.36E-04	0	0.00E+00	10	3.43E-02	5.28E-03

References

(1) Hardacre, C.; Holbrey, J. D.; Mullan, C. L.; Nieuwenhuyzen, M.; Reichert, W. M.; Seddon, K. R.; Teat, S. J. Ionic liquid characteristics of 1-alkyl-n-cyanopyridinium and 1-alkyl-n-(trifluoromethyl)pyridinium salts. *New J. Chem.* **2008**, *32*, 1953–1967.

(2) Bonhote, P.; Dias, A.-P.; Papageorgiou, N.; Kalyanasundaram, K.; Grätzel, M. Hydrophobic, highly conductive ambient-temperature molten salts. *Inorg. Chem.* **1996**, *35*, 1168–1178.

(3) Anthony, J. L.; Anderson, J. L.; Maginn, E. J.; Brennecke, J. F. Anion Effects on Gas Solubility in Ionic Liquids. *J. Phys. Chem. B* **2005**, *109*, 6366–6374.

(4) Xing, H.; Zhao, X.; Li, R.; Yang, Q.; Su, B.; Bao, Z.; Yang, Y.; Ren, Q. Improved efficiency of ethylene/ethane separation using a symmetrical dual nitrile-functionalized ionic liquid. *ACS Sustain. Chem. Eng.* **2013**, *1*, 1357–1363.

(5) Zhang, Q.; Li, Z.; Zhang, J.; Zhang, S.; Zhu, L.; Yang, J.; Zhang, X.; Deng, Y. Physicochemical properties of nitrile-functionalized ionic liquids. *J. Phys. Chem. B* **2007**, *111*, 2864–2872.

(6) Palgunadi, J.; Kim, H. S.; Lee, J. M.; Jung, S. Ionic liquids for acetylene and ethylene separation: Material selection and solubility investigation. *Chem. Eng. Process. Process Intensif.* **2010**, *49*, 192–198.

(7) Moura, L.; Mishra, M.; Bernales, V.; Fuentealba, P.; Padua, A. A.; Santini, C. C.; Costa Gomes, M. F. Effect of unsaturation on the absorption of ethane and ethylene in imidazolium-based ionic liquids. *J. Phys. Chem. B* **2013**, *117*, 7416–7425.
